# Bounding the Inefficiency of Compromise in Opinion Formation

**DOI:** 10.1007/s00453-021-00892-x

**Published:** 2021-11-24

**Authors:** Ioannis Caragiannis, Panagiotis Kanellopoulos, Alexandros A. Voudouris

**Affiliations:** 1 Department of Computer Science, Aarhus University, Aarhus, Denmark; 2 School of Computer Science and Electronic Engineering, University of Essex, Colchester, UK

**Keywords:** Opinion formation, Nash equilibrium, Price of anarchy

## Abstract

Social networks on the Internet have seen an enormous growth recently and play a crucial role in different aspects of today’s life. They have facilitated information dissemination in ways that have been beneficial for their users but they are often used strategically in order to spread information that only serves the objectives of particular users. These properties have inspired a revision of classical opinion formation models from sociology using game-theoretic notions and tools. We follow the same modeling approach, focusing on scenarios where the opinion expressed by each user is a compromise between her internal belief and the opinions of a small number of neighbors among her social acquaintances. We formulate simple games that capture this behavior and quantify the inefficiency of equilibria using the well-known notion of the price of anarchy. Our results indicate that compromise comes at a cost that strongly depends on the neighborhood size.

## Introduction

*Opinion formation* has been the subject of much research in sociology, economics, physics, and epidemiology. Due to the widespread adoption of the Internet and the subsequent blossoming of social networks, it has recently attracted the interest of researchers in computer science at large (e.g., see [3, 8, 25–28]).

An influential model that captures the adoption of opinions in a social context has been proposed by Friedkin and Johnsen [17]. According to this, each individual has an internal belief on an issue and publicly expresses a (possibly different) opinion; internal beliefs and public opinions are modeled as real numbers. In particular, the opinion that an individual expresses follows by averaging between her internal belief and the opinions expressed by her social acquaintances. Recently, Bindel et al. [8] show that this behavior can be explained through a game-theoretic lens: averaging between the internal belief of an individual and the opinions in her social circle is simply a *strategy* that minimizes an implicit cost for the individual.

Bindel et al. [8] use a quadratic function to define this cost. Specifically, this function is equal to the total squared distance of the opinion that the individual expresses from her belief and the opinions expressed in her social circle. In a sense, this behavior leads to opinions that follow the majority of her social acquaintances. Bindel et al. [8] consider a static snapshot of the social network. In contrast, Bhawalkar et al. [6] implicitly assume that the opinion of an individual depends on a small number of acquaintances only, her *neighbors*. So, in their model, opinion formation *co-evolves* with the neighborhood for each individual: her neighborhood consists of those who have opinions similar to her belief. Then, the opinion expressed is assumed to minimize the same cost function used by Bindel et al. [8], taking into account the neighborhood instead of the whole social circle.

We follow the co-evolutionary model of Bhawalkar et al. [6], but we deviate from their cost definition and instead consider individuals that seek to *compromise* with their neighbors. Hence, we assume that each individual aims to minimize the *maximum* distance of her expressed opinion from her belief and each of her neighbors’ opinion. As in [6], we assume that opinion formation co-evolves with the social network. Each individual’s neighborhood consists of the *k* other individuals with the closest opinions to her belief. Naturally, these modeling decisions lead to the definition of strategic games, which we call *k*-*compromising opinion formation* (or, simply, *k*-COF) games. Each individual is a (cost-minimizing) player with the opinion expressed as her strategy.

### Technical Contribution

We study questions related to the existence, computational complexity, and quality of equilibria in *k*-COF games. We begin by proving several properties about the geometric structure of opinions and beliefs at pure Nash equilibria, i.e., in states of the game where each player minimizes her individual cost assuming that the remaining players will not change their opinions, and, thus, has no incentive to deviate by expressing a different opinion.

Using these structural properties we show that there exist simple *k*-COF games that do not admit pure Nash equilibria. Furthermore, we prove that even in games where equilibria do exist, their quality may be suboptimal in terms of the *social cost*, i.e., the total cost experienced by all players. To quantify this inefficiency, we show that the optimistic measure known as the *price of stability* (introduced by Anshelevich et al. [1]) which, informally, is defined as the ratio of the minimum social cost achieved at any pure Nash equilibrium to the minimum social cost at any possible state of the game, grows linearly with *k*.

For the special case of 1-COF games, we show that each such game admits a representation as a directed acyclic graph, in which every pure Nash equilibrium corresponds to a path between two designated nodes. Hence, the problems of computing the best or worst (in terms of the social cost) pure Nash equilibrium (or even of computing whether such an equilibrium exists) are equivalent to simple path computations that can be performed in polynomial time.

For general *k*-COF games, we quantify the inefficiency of the worst-case pure Nash equilibria by bounding the pessimistic measure known as the *price of anarchy* (introduced by Koutsoupias and Papadimitriou [24]) which, informally, is defined as the ratio of the maximum social cost achieved at any pure Nash equilibrium to the minimum possible social cost at any state of the game. Specifically, we present upper and lower bounds on the price of anarchy of *k*-COF games (with respect to both pure and mixed Nash equilibria) that suggest a linear dependence on *k*. Our upper bound on the price of anarchy exploits, in a non-trivial way, linear programming duality in order to lower-bound the optimal social cost. For the fundamental case of 1-COF games, we obtain a tight bound of 3 using a particular charging scheme in the analysis. Our contribution is summarized in Table 1.

**Table 1:** Summary of our results for *k*-COF games

*k*	PoA	MPoA	PoS	Existence/complexity
1	3 (Theorems 14, 15)	$\ge 6$	$\ge 17/15$	PNE may not exist for any *k* (Theorem 6)
		(Theorem 16)	(Theorem 8)	Best and worst PNE is in ${{{\mathcal {P}}}}$ (Thm. 10)
2	$\le 12$ (Theorem 12)	$\ge 24/5$	$\ge 8/7$	Open question: Is computing a PNE
	$\ge 18/5$ (Theorem 17)	(Theorem 18)	(Theorem 9)	in ${{{\mathcal {P}}}}$ when $k\ge 2$?
$\ge 3$	$\le 4(k+1)$ (Theorem 12)	$\ge k+2$	$\ge \frac{k+1}{3}$	
	$\ge k+1$ (Theorem 17)	(Theorem 18)	(Theorem 7)	

The table presents our bounds on the price of anarchy over pure Nash equilibria (PoA) and mixed Nash equilibria (MPoA), on the price of stability (PoS) as well as the existence and complexity of pure Nash equilibria (PNE). Clearly, any upper bound on PoA is also an upper bound on PoS

### Related Work

DeGroot [12] proposed a framework that models the opinion formation process, where each individual updates her opinion based on a weighted averaging procedure. Subsequently, Friedkin and Johnsen [17] refined the model by assuming that each individual has a private belief and expresses a (possibly different) public opinion that depends on her belief and the opinions of people to whom she has social ties. More recently, Bindel et al. [8] studied this model and proved that, for the setting where beliefs and opinions are in [0, 1], the repeated averaging process leads to an opinion vector that can be thought of as the unique equilibrium in a corresponding opinion formation game.

Deviating from the assumption that opinions depend on the whole social circle, Bhawalkar et al. [6] consider co-evolutionary opinion formation games, where as opinions evolve so does the neighborhood of each person. This model is conceptually similar to previous ones that have been studied by Hegselmann and Krause [19], and Holm and Newman [20]. Both Bindel et al. [8] and Bhawalkar et al. [6] show constant bounds on the price of anarchy of the games that they study. In contrast, the modified cost function we use in order to model compromise yields considerably higher price of anarchy.

A series of recent papers from the EconCS community consider discrete models with binary opinions. Chierichetti et al. [10] consider discrete preference games, where beliefs and opinions are binary and study questions related to the price of stability. For these games, Auletta et al. [2, 4] characterize the social networks where the belief of the minority can emerge as the opinion of the majority, while in [5] they examine the robustness of such results to variants of the model. Auletta et al. [3] generalize discrete preference games so that players are not only interested in agreeing with their neighbors and more complex constraints can be used to represent the players’ preferences. Bilò et al. [7] extend co-evolutionary formation games to the discrete setting. Other models assume that opinion updates depend on the entire social circle of each individual, who consults a small random subset of social acquaintances; see the recent paper by Fotakis et al. [16] and the survey of Mossel and Tamuz [25].

When there are more than one issues to be discussed, Jia et al. [21] propose and analyze the DeGroot-Friedkin model for the evolution of an influence network between individuals who form opinions on a sequence of issues, while Xu et al. [29] introduce a modification to the DeGroot-Friedkin model so that each individual may recalculate the weight given to her opinion, i.e., her self-confidence, after the discussion of each issue.

Another line of research considers how fast a system converges to a stable state. In this spirit, Etesami and Basar [13] consider the dynamics of the Hegselmann-Krause model [19], where opinions and neighborhoods co-evolve, and study the termination time in finite dimensions under different settings. Similarly, Ferraioli et al. [14] study the speed of convergence of decentralized dynamics in finite opinion games, where players have only a finite number of opinions available. Ferraioli and Ventre [15] consider the role of social pressure towards consensus in opinion games and provide tight bounds on the speed of convergence for the important special case where the social network is a clique.

Das et al. [11] perform a set of online user studies and argue that widely studied theoretical models do not completely explain the experimental results obtained. Hence, they introduce an analytical model for opinion formation and present preliminary theoretical and simulation results on the convergence and structure of opinions when users iteratively update their respective opinions according to the new model.

Chazelle [9] analyzes influence systems, where each individual observes the location of her neighbors and moves accordingly, and presents an algorithmic calculus for studying such systems. Kempe et al. [22] present a novel model of cultural dynamics and study the interplay between selection and influence. Their results include an almost complete characterization of stable outcomes and guaranteed convergence from all starting states. Gomez-Rodriguez et al. [18] consider network diffusion and contagion propagation. Their goal is to infer an unknown network over which contagion propagated, tracing paths of diffusion and influence. Finally, Kempe et al. [23] study the optimization problem for influence maximization in a social networks, where each individual may decide to adopt an idea or an innovation depending on how many of her neighbors already do. The goal is to select an initial seed set of early adopters so that the number of adopters is maximized.

In spite of the extensive related literature on opinion formation in many different disciplines, our model introduces a novel cost function that, we believe, presents an interesting alternative on how individuals tend to compromise. In particular, while [12, 17] rely on a weighted average of a player’s belief and her neighbors’ opinions, our model suggests that, apart from the player’s belief, the two most polarized opinions among those of her neighbors are the most important ones, and thus attempts to minimize the maximum opinion dissonance. For example, if all neighbors have roughly the same opinion that is relatively distant from the player’s belief, the average leads to an opinion very close to that of her neighbors, thus essentially disregarding the player’s belief; in contrast, our model achieves a more reasonable compromise by leading to an opinion that is the midpoint between the player’s belief and her neighbors’ opinion. On the other extreme, when most neighbors express opinions close to the player’s belief but there are also a few of them with a quite distant opinion, our model interprets compromise as a behavior that favors the diversity of opinions and beliefs.

### Roadmap

We begin with preliminary definitions and notation in Sect. 2. Then, in Sect. 3 we present several structural properties of pure Nash equilibria, while Sect. 4 is devoted to the existence and the price of stability of these equilibria. In Sect. 5, we present an algorithm that determines whether pure Nash equilibria exist in a 1-COF game, and, in addition, computes the best and worst such equilibria, when they exist. In Sects. 6 and 7 we prove upper bounds on the price of anarchy of *k*-COF and 1-COF games, respectively, while Sect. 8 contains our lower bounds. We conclude in Sect. 9 with a discussion of open problems and possible extensions of our work.

## Definitions and Notation

A compromising opinion formation game defined by the *k* nearest neighbors (henceforth, called *k*-COF game) is played by a set of *n* players whose beliefs lie on the line of real numbers. Let ${\mathbf {s}}=(s_1,s_2,\dots ,s_n) \in {\mathbb {R}}^n$ be the vector containing the players’ beliefs such that $s_i \le s_{i+1}$ for each $i \in [n-1]$. Let ${\mathbf {z}}=(z_1,z_2,\dots ,z_n) \in {\mathbb {R}}^n$ be a vector containing the (deterministic or randomized) opinions expressed by the players; these opinions define a state of the game. We denote by ${\mathbf {z}}_{-i}$ the opinion vector obtained by removing $z_i$ from ${\mathbf {z}}$. In an attempt to simplify notation, we omit *k* from all relevant definitions.

Given vector ${\mathbf {z}}$ (or a realization of it in case ${\mathbf {z}}$ contains randomized opinions), we define the neighborhood $N_i({\mathbf {z}}, {\mathbf {s}})$ of player *i* to be the set of *k* players whose opinions are the closest to the belief of player *i* breaking ties arbitrarily (but consistently). For each player *i*, we define $I_i({\mathbf {z}}, {\mathbf {s}})$ as the shortest interval of the real line that includes the following points: the belief $s_i$, the opinion $z_i$, and the opinion $z_j$ for each player $j \in N_i({\mathbf {z}}, {\mathbf {s}})$. Furthermore, let $\ell _i({\mathbf {z}}, {\mathbf {s}})$ and $r_i({\mathbf {z}}, {\mathbf {s}})$ be the players with the leftmost and rightmost point in $I_i({\mathbf {z}}, {\mathbf {s}})$, respectively. For example, $\ell _i({\mathbf {z}},{\mathbf {s}})$ can be equal to either player *i* or some player $j\in N_i({\mathbf {z}},{\mathbf {s}})$, depending on whether the leftmost point of $I_i({\mathbf {z}},{\mathbf {s}})$ is $s_i$, $z_i$, or $z_j$. To further simplify notation, we will frequently use $\ell (i)$ and *r*(*i*) instead of $\ell _i({\mathbf {z}},{\mathbf {s}})$ and $r_i({\mathbf {z}}, {\mathbf {s}})$ when ${\mathbf {z}}$ and ${\mathbf {s}}$ are clear from the context. In the following, we present the relevant definitions for the case of possibly randomized opinion vectors; clearly, these can be simplified whenever ${\mathbf {z}}$ consists entirely of deterministic opinions.

Given a *k*-COF game with belief vector ${\mathbf {s}}$, the cost that player *i* experiences at the state of the game defined by an opinion vector ${\mathbf {z}}$ is

1 $$\begin{aligned} \nonumber {\mathbb {E}}[{{\,\mathrm{cost}\,}}_i({\mathbf {z}}, {\mathbf {s}})]&= {\mathbb {E}}\left[ \max _{j \in N_i({\mathbf {z}},{\mathbf {s}})}\bigg \{ |z_i-s_i|, |z_j-z_i| \bigg \}\right] \\&= {\mathbb {E}}\left[ \max \bigg \{ |z_i-s_i|, |z_{r_i({\mathbf {z}},{\mathbf {s}})}-z_i|, |z_i-z_{\ell _i({\mathbf {z}},{\mathbf {s}})}| \bigg \}\right] . \end{aligned}$$

For the special case of 1-COF games, we denote by $\sigma _i({\mathbf {z}},{\mathbf {s}})$ (or $\sigma (i)$ when ${\mathbf {z}}$ and ${\mathbf {s}}$ are clear from the context) the player (other than *i*) whose opinion is closest to the belief $s_i$ of player *i*; notice that $\sigma (i)$ is the only member of $N_i({\mathbf {z}},{\mathbf {s}})$. In this case, the cost of player *i* can be simplified as

2 $$\begin{aligned} {\mathbb {E}}[{{\,\mathrm{cost}\,}}_i({\mathbf {z}}, {\mathbf {s}})] = {\mathbb {E}}\left[ \max \bigg \{ |z_i-s_i|, |z_{\sigma _i({\mathbf {z}},{\mathbf {s}})}-z_i| \bigg \}\right] . \end{aligned}$$

We say that an opinion vector ${\mathbf {z}}$ consisting entirely of deterministic opinions is a *pure Nash equilibrium* if no player *i* has an incentive to unilaterally deviate to a deterministic opinion $z_i'$ in order to decrease her cost, i.e.,

$$\begin{aligned} {{\,\mathrm{cost}\,}}_i({\mathbf {z}}, {\mathbf {s}}) \le {{\,\mathrm{cost}\,}}_i((z_i',{\mathbf {z}}_{-i}), {\mathbf {s}}), \end{aligned}$$

where by $(z_i',{\mathbf {z}}_{-i})$ we denote the opinion vector in which player *i* chooses the opinion $z_i'$ and all other players choose the opinions they have according to vector ${\mathbf {z}}$. Similarly, a possibly randomized opinion vector ${\mathbf {z}}$ is a *mixed Nash equilibrium* if for any player *i* and any deviating deterministic opinion $z_i'$ we have

$$\begin{aligned} {\mathbb {E}}[{{\,\mathrm{cost}\,}}_i({\mathbf {z}}, {\mathbf {s}})] \le {\mathbb {E}}_{{\mathbf {z}}_{-i}}[{{\,\mathrm{cost}\,}}_i((z_i',{\mathbf {z}}_{-i}), {\mathbf {s}})]. \end{aligned}$$

Let $\text {PNE}({\mathbf {s}})$ and $\text {MNE}({\mathbf {s}})$ denote the sets of pure and mixed Nash equilibria, respectively, of the *k*-COF game with belief vector ${\mathbf {s}}$.

The *social cost* of an opinion vector ${\mathbf {z}}$ is the total cost experienced by all players, i.e.,

$$\begin{aligned} {\mathbb {E}}[{{\,\mathrm{\text {SC}}\,}}({\mathbf {z}}, {\mathbf {s}})] = \sum _{i=1}^n{ {\mathbb {E}}[{{\,\mathrm{cost}\,}}_i({\mathbf {z}}, {\mathbf {s}})] }. \end{aligned}$$

Let ${\mathbf {z}}^*({\mathbf {s}})$ be a deterministic opinion vector that minimizes the social cost for the given *k*-COF game with belief vector ${\mathbf {s}}$; we will refer to it as an *optimal* opinion vector for ${\mathbf {s}}$.

The *price of anarchy* (PoA) over pure Nash equilibria of a particular *k*-COF game with belief vector ${\mathbf {s}}$ is defined as the ratio between the social cost of its *worst* (in terms of the social cost) pure Nash equilibrium and the optimal social cost, i.e.,

$$\begin{aligned} \text {PoA}({\mathbf {s}}) = \sup _{{\mathbf {z}}\in \text {PNE}({\mathbf {s}})} \frac{{{\,\mathrm{\text {SC}}\,}}({\mathbf {z}}, {\mathbf {s}})}{{{\,\mathrm{\text {SC}}\,}}({\mathbf {z}}^*({\mathbf {s}}), {\mathbf {s}})}. \end{aligned}$$

The *price of stability* (PoS) over pure Nash equilibria of the *k*-COF game with belief vector ${\mathbf {s}}$ is defined as the ratio between the social cost of the *best* pure Nash equilibrium (in terms of social cost) and the optimal social cost, i.e.,

$$\begin{aligned} \text {PoS}({\mathbf {s}}) = \inf _{{\mathbf {z}}\in \text {PNE}({\mathbf {s}})} \frac{{{\,\mathrm{\text {SC}}\,}}({\mathbf {z}}, {\mathbf {s}})}{{{\,\mathrm{\text {SC}}\,}}({\mathbf {z}}^*({\mathbf {s}}), {\mathbf {s}})}. \end{aligned}$$

Similarly, the price of anarchy and the price of stability over mixed Nash equilibria of a *k*-COF game with belief vector ${\mathbf {s}}$ are defined as

$$\begin{aligned} \text {MPoA}({\mathbf {s}}) = \sup _{{\mathbf {z}}\in \text {MNE}({\mathbf {s}})} \frac{{\mathbb {E}}[{{\,\mathrm{\text {SC}}\,}}({\mathbf {z}}, {\mathbf {s}})]}{{{\,\mathrm{\text {SC}}\,}}({\mathbf {z}}^*({\mathbf {s}}), {\mathbf {s}})} \end{aligned}$$

and

$$\begin{aligned} \text {MPoS}({\mathbf {s}}) = \inf _{{\mathbf {z}}\in \text {MNE}({\mathbf {s}})} \frac{{\mathbb {E}}[{{\,\mathrm{\text {SC}}\,}}({\mathbf {z}}, {\mathbf {s}})]}{{{\,\mathrm{\text {SC}}\,}}({\mathbf {z}}^*({\mathbf {s}}), {\mathbf {s}})}, \end{aligned}$$

respectively.

Then, the price of anarchy and the price of stability of *k*-COF games, for a fixed *k*, are defined as the supremum of $\text {PoA}({\mathbf {s}})$ and $\text {PoS}({\mathbf {s}})$ over all belief vectors ${\mathbf {s}}$, respectively.

We conclude this section with an example.

### Example 1

**Fig. 1:**
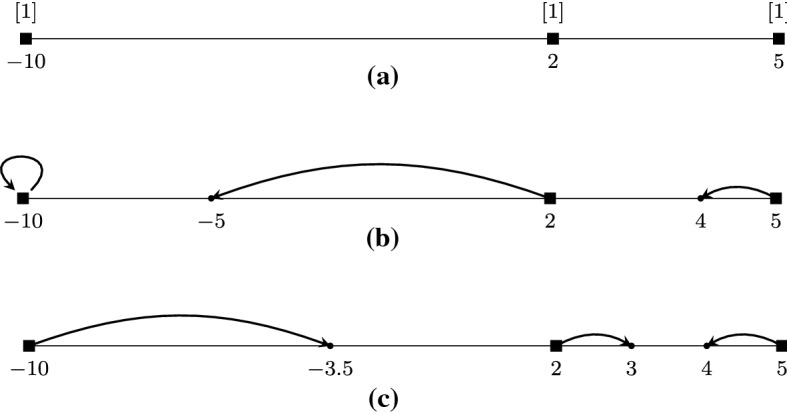
The game examined in Example 1. **a** Illustration of the belief vector ${\mathbf {s}}= (-10, 2, 5)$. The black squares correspond to player beliefs. The notation [*x*] is used to denote the number of players that have the same beliefs; here we have only one player per belief. **b** Illustration of the opinion vector ${\mathbf {z}}=(-10,-5,4)$. The dots correspond to player opinions and each arrow connects the belief of a player to her opinion. **c** Illustration of the equilibrium opinion vector ${\mathbf {z}}'=(-3.5,3,4)$

Consider the 1-COF game with three players and belief vector ${\mathbf {s}}= (-10,2,5)$ which is depicted in Fig. 1a. For simplicity, we will refer to the players as left ($\ell$), middle (*m*), and right (*r*).

Let us examine the opinion vector ${\mathbf {z}}= (-10,-5,4)$ which is depicted in Fig. 1b. We have that $\sigma (\ell ) = m$ since the opinion $z_m=-5$ of the middle player is closer to the belief $s_\ell =-10$ of the left player than the opinion $z_r=4$ of the right player. Therefore, the cost of the left player is ${{\,\mathrm{cost}\,}}_\ell ({\mathbf {z}},{\mathbf {s}}) = \max \{|-10+10|,|-10+5|\} = 5$. Similarly, the neighbors of the middle and right players are $\sigma (m) = r$ and $\sigma (r) = m$, while their costs are ${{\,\mathrm{cost}\,}}_m({\mathbf {z}},{\mathbf {s}}) = \max \{2+5,4+5\} = 9$ and ${{\,\mathrm{cost}\,}}_r({\mathbf {z}},{\mathbf {s}}) = \max \{5-4,4+5\} = 9$, respectively. The social cost is ${{\,\mathrm{\text {SC}}\,}}({\mathbf {z}},{\mathbf {s}}) = 23$.

Now, consider the alternative pure Nash equilibrium opinion vector ${\mathbf {z}}' = (-3.5,3,4)$ which is depicted in Fig. 1c. Observe that even though ${\mathbf {z}}' \ne {\mathbf {z}}$, each player has the same neighbor as in ${\mathbf {z}}$ and no player has an incentive to deviate in order to decrease her cost. Indeed, let us focus on the middle player for whom it is $\sigma (m) = r$. Her opinion is in the midpoint of the interval defined by her belief $s_m = 2$ and the opinion $z_r' = 4$ of the right player. Hence, this opinion minimizes her cost by minimizing the maximum between the distance from her belief and the distance from the opinion of the right player. It is easy to verify that the same holds for the left and right players. The player costs are now 6.5, 1, and 1, respectively, yielding a social cost of 8.5. $\square$

## Some Properties About Equilibria

We devote this section to proving several interesting properties of pure Nash equilibria; these will be useful in the following. The first one is obvious due to the definition of the cost function.

### Lemma 1

In any pure Nash equilibrium ${\mathbf {z}}$ of a *k*-COF game with belief vector ${\mathbf {s}}$, the opinion of any player *i* lies in the midpoint of the interval $I_i({\mathbf {z}}, {\mathbf {s}})$.

The next lemma allows us to argue about the order of player opinions in a pure Nash equilibrium ${\mathbf {z}}$.

### Lemma 2

In any pure Nash equilibrium ${\mathbf {z}}$ of a *k*-COF game with belief vector ${\mathbf {s}}$, it holds that $z_i\le z_{i+1}$ for any $i \in [n-1]$ such that $s_i < s_{i+1}$.

### Proof

For the sake of contradiction, let us assume that $z_{i+1}<z_i$ for a pair of players *i* and $i+1$ with $s_i < s_{i+1}$. Then, it cannot be the case that the leftmost endpoint of the interval $I_i({\mathbf {z}},{\mathbf {s}})$ of player *i* is at the left of (or coincides with) the leftmost endpoint of interval $I_{i+1}({\mathbf {z}},{\mathbf {s}})$ of player $i+1$ and the rightmost endpoint of $I_i({\mathbf {z}},{\mathbf {s}})$ is at the left of (or coincides with) the rightmost endpoint of $I_{i+1}({\mathbf {z}},{\mathbf {s}})$. In other words, it cannot be the case that $\min \{s_i,z_{\ell (i)}\}\le \min \{s_{i+1},z_{\ell (i+1)}\}$ and $\max \{s_i,z_{r(i)}\} \le \max \{s_{i+1},z_{r(i+1)}\}$ hold simultaneously. Since, by Lemma 1, points $z_i$ and $z_{i+1}$ lie in the midpoint of the corresponding intervals, we would have $z_i\le z_{i+1}$, contradicting our assumption.

**Fig. 2:**
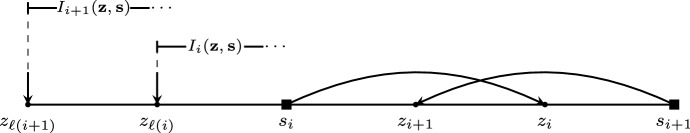
An example of the argument used in the proof of Lemma 2

So, at least one of the two inequalities between the interval endpoints above must not hold. In the following, we assume that $\min \{s_i,z_{\ell (i)}\}>\min \{s_{i+1},z_{\ell (i+1)}\}$ (the case where $\max \{s_i,z_{r(i)}\} >\max \{s_{i+1},z_{r(i+1)}\}$ is symmetric). This assumption implies that $z_{\ell (i+1)}<s_i < s_{i+1}$ (i.e., $\min \{s_{i+1},z_{\ell (i+1)}\} = z_{\ell (i+1)}$), and, subsequently, that $z_{\ell (i+1)}<z_{\ell (i)}$. In words, player $\ell (i+1)$ does not belong to interval $I_i({\mathbf {z}},{\mathbf {s}})$. Furthermore, since $z_{\ell (i+1)} < s_{i+1}$, and as (by Lemma 1) $z_{i+1}$ lies in the midpoint of $I_{i+1}({\mathbf {z}},{\mathbf {s}})$, we also have that the leftmost endpoint of interval $I_{i+1}({\mathbf {z}},{\mathbf {s}})$ cannot belong to player $i+1$, i.e., $\ell (i+1)\not =i+1$. An example of the relative ordering of points (beliefs and opinions), after assuming that $z_{i+1} < z_i$ and $\min \{s_i,z_{\ell (i)}\}>\min \{s_{i+1},z_{\ell (i+1)}\}$ is depicted in Fig. 2.

Since $\ell (i+1)$ does not belong to $I_i({\mathbf {z}},{\mathbf {s}})$, there are at least *k* players different than $\ell (i+1)$ and *i* that have opinions at distance at most $s_i-z_{\ell (i+1)}$ from belief $s_i$. Since $s_i < s_{i+1}$ and $z_{\ell (i+1)} < z_{\ell (i)}$, all these players are also at distance strictly less than $s_{i+1}-z_{\ell (i+1)}$ from belief $s_{i+1}$. This contradicts the fact that the opinion of player $\ell (i+1)$ is among the *k* closest opinions to $s_{i+1}$. $\square$

In the following, in any pure Nash equibrium ${\mathbf {z}}$, we assume that $z_i\le z_{i+1}$ for any $i \in [n-1]$. This follows by Lemma 2 when $s_i < s_{i+1}$ and by a convention for the identities of players with identical belief.

In addition to the ordering of opinions in a pure Nash equilibrium, we can also specify the range of neighborhoods (in Lemma 3) and opinions (in Lemma 4).

### Lemma 3

Let ${\mathbf {z}}$ be a pure Nash equilibrium of a *k*-COF game with belief vector ${\mathbf {s}}$. Then, for each player *i*, there exists *j* with $i-k\le j\le i$ such that $I_i({\mathbf {z}},{\mathbf {s}})$ is the shortest interval that contains the opinions $z_j, z_{j+1}, ..., z_{j+k}$ and belief $s_i$.

### Proof

If $I_i({\mathbf {z}},{\mathbf {s}})$ consists of a single point, the lemma follows trivially by the definition of the neighborhood and Lemma 2 since at least $k+1$ consecutive players including *i* should have opinions in $I_i({\mathbf {z}},{\mathbf {s}})$. Otherwise, by Lemma 2, the lemma is true if there is at most one opinion in each of the left and the right boundary of $I_i({\mathbf {z}},{\mathbf {s}})$; in this case, there are exactly $k+1$ consecutive players including player *i* with opinions in $I_i({\mathbf {z}},{\mathbf {s}})$.

In the following, we handle the subtleties that may arise due to tie-breaking at the boundaries of $I_i({\mathbf {z}},{\mathbf {s}})$. Let $Y_\ell$ and $Y_r$ be the set of players with opinions at the leftmost and the rightmost point of $I_i({\mathbf {z}},{\mathbf {s}})$, respectively. From Lemma 1, player *i* belongs neither to $Y_\ell$ nor to $Y_r$. Now consider the following set of players: the $|Y_\ell \cap N_i({\mathbf {z}},{\mathbf {s}})|$ players with highest indices from $Y_\ell$, the $|Y_r\cap N_i({\mathbf {z}},{\mathbf {s}})|$ players with lowest indices from $Y_r$ and all players with opinions that lie strictly in $I_i({\mathbf {z}},{\mathbf {s}})$. Due to the definition of $N_i({\mathbf {z}},{\mathbf {s}})$ and by Lemma 2, there are $k+1$ players in this set, including player *i*, with consecutive indices. $\square$

In the following, irrespective of how ties are actually resolved, we assume that $N_i({\mathbf {z}},{\mathbf {s}})\cup \{i\}$ consists of $k+1$ players with consecutive indices. This does not affect the cost of player *i* at equilibrium in the proofs of our upper bounds (since, by Lemma 3, the interval defined is exactly the same), while our lower bound constructions are defined carefully so that the results hold no matter how ties are actually resolved.

### Lemma 4

Let ${\mathbf {z}}$ be a pure Nash equilibrium of a *k*-COF game with belief vector ${\mathbf {s}}$. Then, for each player *i*, it holds that $s_{\ell (i)} \le z_i\le s_{r(i)}$.

### Proof

**Fig. 3:**
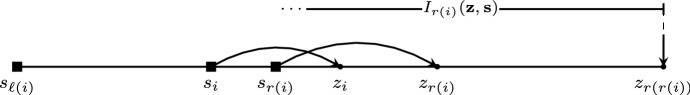
An example of the argument used in the proof of Lemma 4

Since $N_i({\mathbf {z}},{\mathbf {s}}) \cup \{i\}$ consists of $k+1$ players with consecutive indices, we have that $s_{\ell (i)} \le s_i \le s_{r(i)}$. For the sake of contradiction, let us assume that $s_{\ell (i)}\le s_{r(i)}<z_i$ for some player *i* (the case where $z_i$ lies at the left of $s_{\ell (i)}$ is symmetric). Since $s_i\le s_{r(i)} < z_i$ and as $z_i$ is at the midpoint of $I_i({\mathbf {z}}, {\mathbf {s}})$, it holds that $z_{r(i)}>z_i$ (i.e., $r(i) \ne i$). Also, since $z_{r(i)}>z_i>s_{r(i)}$, and because $z_{r(i)}$ is in the midpoint of $I_{r(i)}({\mathbf {z}}, {\mathbf {s}})$, it holds that $z_{r(r(i))} > z_{r(i)}$ and, by Lemma 2, $r(r(i))>r(i)$; see Fig. 3 for an example of the relative ordering of points (beliefs and opinions) when assuming that $s_{r(i)} < z_i$.

We now claim that $\ell (i) \notin N_{r(i)}({\mathbf {z}}, {\mathbf {s}})$. Assume otherwise that $\ell (i)\in N_{r(i)}({\mathbf {z}}, {\mathbf {s}})$. By definition, $r(r(i))\in N_{r(i)}({\mathbf {z}}, {\mathbf {s}})$. Then, Lemma 2 implies that any player *j*, different than *r*(*i*), with $\ell (i)<j<r(r(i))$ is also in $N_{r(i)}({\mathbf {z}}, {\mathbf {s}})$. Hence, $N_{r(i)}({\mathbf {z}},{\mathbf {s}})$ contains at least the $k-1$ players in $N_i({\mathbf {z}},{\mathbf {s}})\setminus \{r(i)\}$, as well as players *i* and *r*(*r*(*i*)). This, however, contradicts the fact that $|N_{r(i)}({\mathbf {z}}, {\mathbf {s}})| = k$. Therefore, player $\ell (i)$ is not among the *k* nearest neighbors of *r*(*i*).

So, we obtain that

$$\begin{aligned} z_{r(r(i))} - s_{r(i)}&> z_{r(i)} - s_{r(i)}> z_{r(i)} - z_i = z_i - \min \{s_i, z_{\ell (i)}\} \\&> s_{r(i)} - \min \{s_i, z_{\ell (i)}\} \ge s_{r(i)} - z_{\ell (i)}. \end{aligned}$$

If $z_{\ell (i)} > s_{r(i)}$ (i.e, $z_{\ell (i)}$ is at the right of $s_{r(i)}$), then since, by Lemma 2, $z_{\ell (i)}\le z_{r(r(i))}$ and $r(r(i)) \in N_{r(i)}({\mathbf {z}}, {\mathbf {s}})$, we obtain that $\ell (i) \in N_{r(i)}({\mathbf {z}}, {\mathbf {s}})$ as well; a contradiction. Otherwise, the above inequality yields that $z_{r(r(i))} - s_{r(i)} > s_{r(i)} - z_{\ell (i)}\ge 0$ (i.e., the distance of $s_{r(i)}$ from $z_{r(r(i))}$ is strictly higher than the distance of $s_{r(i)}$ from $z_{\ell (i)}$), and, again, we obtain a contradiction to the fact that $\ell (i)\notin N_{r(i)}({\mathbf {z}}, {\mathbf {s}})$ and $r(r(i)) \in N_{r(i)}({\mathbf {z}}, {\mathbf {s}})$. $\square$

## Existence and Quality of Equilibria

Our first technical contribution is a negative statement: pure Nash equilibria may not exist for any *k* (Theorem 6). Then, we show that even in *k*-COF games that admit pure Nash equilibria, the best equilibrium may be inefficient; in other words, the price of stability is strictly greater than 1 for any value of *k*, and, actually, depends linearly on *k*. These results appear in Theorems 7, 8, and 9.

### Existence of Equilibria

We begin with a technical lemma. The lemma essentially presents necessary conditions so that a particular set of neighborhoods, and corresponding intervals, may coexist in a pure Nash equilibrium.

#### Lemma 5

Consider a *k*-COF game and any three players *a*, *b*, *c* with beliefs $s_a\le s_b\le s_c$, respectively. For any pure Nash equilibrium ${\mathbf {z}}$ where $I_a({\mathbf {z}}, {\mathbf {s}}) = [s_a, z_b]$, $I_b({\mathbf {z}}, {\mathbf {s}}) = [s_b, z_c]$ and $I_c({\mathbf {z}}, {\mathbf {s}}) = [z_b, s_c]$, it must hold that $s_b\ge \frac{3s_a+5s_c}{8}$, while for any pure Nash equilibrium ${\mathbf {z}}$ where $I_a({\mathbf {z}}, {\mathbf {s}}) = [s_a, z_b]$, $I_b({\mathbf {z}}, {\mathbf {s}}) = [z_a, s_b]$ and $I_c({\mathbf {z}}, {\mathbf {s}}) = [z_b, s_c]$, it must hold that $s_b\le \frac{5s_a+3s_c}{8}$.

#### Proof

It suffices to prove the first case; the second case is symmetric. First, observe that if $s_b = s_c$, the claim holds trivially; so, we assume that $s_b<s_c$. Since $I_b({\mathbf {z}}, {\mathbf {s}}) = [s_b, z_c]$ and $I_c({\mathbf {z}}, {\mathbf {s}}) = [z_b, s_c]$, by Lemma 1 it holds that $z_b = (s_b+z_c)/2$ and $z_c = (z_b+s_c)/2$ which yield that $z_b = s_b+\frac{s_c-s_b}{3}$ and $z_c = s_b+\frac{2(s_c-s_b)}{3}$. Hence, we obtain that

3 $$\begin{aligned} z_c - s_b = \frac{2(s_c-s_b)}{3}. \end{aligned}$$

Similarly, since $I_a({\mathbf {z}}, {\mathbf {s}}) = [s_a, z_b]$, it holds that $z_a = \frac{s_a+z_b}{2}= \frac{3s_a+2s_b+s_c}{6}$ and, therefore, we obtain that

4 $$\begin{aligned} s_b-z_a = \frac{-3s_a+4s_b-s_c}{6}. \end{aligned}$$

Since $I_b({\mathbf {z}}, {\mathbf {s}}) = [s_b, z_c]$, we have that either $a\notin N_b({\mathbf {z}}, {\mathbf {s}})$ or, if $a \in N_b({\mathbf {z}}, {\mathbf {s}})$, it must be $z_a\ge s_b$. In the first case, we have $z_c-s_b\le s_b-z_a$ which, together with (3) and (4), yields that $s_b\ge \frac{3s_a+5s_c}{8}$ as desired. In the latter case, we have $z_a = \frac{3s_a+2s_b+s_c}{6} \ge s_b$, i.e.,

5 $$\begin{aligned} s_b \le \frac{3s_a+s_c}{4}. \end{aligned}$$

Now, observe that $\ell (a)<a$ as, otherwise, it cannot be that $a \in N_b({\mathbf {z}}, {\mathbf {s}})$, $c \in N_b({\mathbf {z}}, {\mathbf {s}})$ but $c \notin N_a({\mathbf {z}}, {\mathbf {s}})$. Furthermore, it must be $\ell (a) \notin N_b({\mathbf {z}}, {\mathbf {s}})$ as $\ell (a) \in N_a({\mathbf {z}}, {\mathbf {s}})$, $c \in N_b({\mathbf {z}},{\mathbf {s}})$, $c\notin N_a({\mathbf {z}},{\mathbf {s}})$, and each neighborhood contains exactly *k* players. So, it holds $s_b - z_{\ell (a)} \ge z_c - s_b$, i.e., as $z_{\ell (a)}\ge s_a$, $s_b-s_a \ge z_c-s_b = \frac{2(s_c-s_b)}{3}$. This gives that

6 $$\begin{aligned} s_b \ge \frac{3s_a+2s_c}{5}, \end{aligned}$$

which, as $s_a<s_c$, contradicts (5). Therefore, it cannot be that $a \in N_b({\mathbf {z}}, {\mathbf {s}})$. $\square$

The proof of the next theorem is inspired by a construction of [6] and exploits Lemma 5.

#### Theorem 6

For any *k*, there exists a *k*-COF game with no pure Nash equilibria.

#### Proof

Consider a *k*-COF game with $2k+1$ players partitioned into three sets called *L*, *M*, and *R*, where *L* and *R* each contain *k* players, while $M = \{m\}$ is a singleton. We set $s_i = 0$ for each $i \in L$, $s_i = 2$ for each $i \in R$, while $s_m = 1-\epsilon$, where $\epsilon < 1/4$ is an arbitrarily small positive constant.

Let us assume that there exists a pure Nash equilibrium ${\mathbf {z}}$. Then, clearly, for any $i \in L$ it must hold that $N_i({\mathbf {z}}, {\mathbf {s}}) = L\setminus \{i\} \cup \{m\}$, and, therefore, $I_i({\mathbf {z}}, {\mathbf {s}}) = [0, z_m]$. Similarly, for any $i \in R$ we have $N_i({\mathbf {z}}, {\mathbf {s}}) = R \setminus \{i\} \cup \{m\}$, and $I_i({\mathbf {z}}, {\mathbf {s}}) = [z_m, 2]$. Now, concerning player *m*, if all her neighbors are in *L*, then, it holds that $I_m({\mathbf {z}}, {\mathbf {s}}) = [z_i, s_m]$ for some $i\in L$. But then, observe that even though the intervals defined above exhibit the structure described in Lemma 5, the belief vector ${\mathbf {s}}$ does not satisfy the corresponding necessary conditions of that lemma as $1-\epsilon > 3/4$; hence, ${\mathbf {z}}$ is not a pure Nash equilibrium. The same reasoning applies in case all of *m*’s neighbors are in *R*.

It remains to consider the case where *m* has at least one neighbor in each of *L* and *R*. By the definition of $I_i({\mathbf {z}}, {\mathbf {s}})$ for $i \in L \cup R$, as stated above, Lemma 1 implies that $z_i = z_m/2$ for any $i\in L$, while $z_i = 1+z_m/2$ for any $i\in R$. Then, Lemma 4 implies that $z_m/2\le s_m = 1-\epsilon$ and $1+z_m/2 \ge s_m$, and, consequently, $I_m({\mathbf {z}}, {\mathbf {s}}) = [z_m/2, 1+z_m/2]$. Again, by Lemma 1 we have that $z_m = \frac{z_m/2+1+z_m/2}{2}$, i.e., $z_m = 1$. But then, we obtain $z_i= 1/2$ for any $i\in L$ and $z_i = 3/2$ for any $i\in R$, which implies that all *k* players in *L* are strictly closer to $s_m$ than any player in *R*; this contradicts the assumption that *m* has neighbors in both *L* and *R*. $\square$

**Fig. 4:**
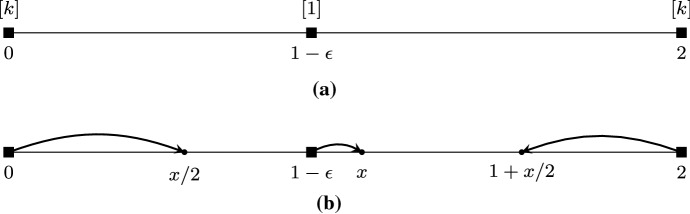
**a** The *k*-COF game considered in the proof of Theorem 6 where the *k* players of set *L* have belief 0, player *m* has $s_m = 1-\epsilon$ and the *k* players of set *R* have belief 2. **b** Lemma 5 implies that there is no pure Nash equilibrium where *m* has neighbors in strictly one of *L*, *R*. In the remaining case, it must hold that $x = 1$, but then all players in *L* are strictly closer to $s_m$ than any player in *R*

An example of the construction used in the proof of Theorem 6 is presented in Fig. 4.

### Price of Stability

We will now prove that the price of stability of *k*-COF games is strictly higher than 1, i.e., there exist games without any efficient pure Nash equilibria (even when they exist). In particular, for any value of *k* we show that there exist rather simple games with price of stability in $\varOmega (k)$.

#### Theorem 7

The price of stability of *k*-COF games, for $k\ge 3$, is at least $(k+1)/3$.

#### Proof

Consider a *k*-COF game with $k+1$ players, where *k* of them have belief 0, while the remaining one has belief 1. Let ${\tilde{{\mathbf {z}}}}$ be the opinion vector where each player has opinion 0. Clearly, ${{\,\mathrm{\text {SC}}\,}}({\tilde{{\mathbf {z}}}}, {\mathbf {s}}) = 1$, and, hence the optimal social cost is at most 1.

Now, consider any pure Nash equilibrium ${\mathbf {z}}$. Since, there are $k+1$ players, the neighborhood of each player includes all remaining ones. Let *x* be the opinion that the player with belief 1 expresses at ${\mathbf {z}}$. By Lemma 4, we have that $x \in [0,1]$, and by Lemma 1, we have that all remaining players must have opinion *x*/2. Therefore, again by Lemma 1, *x* must satisfy the equation $x = (1+x/2)/2$, i.e., $x=2/3$. Therefore, there exists a single pure Nash equilibrium ${\mathbf {z}}$ where all players with belief 0 have opinion 1/3 and the single player with belief 1 has opinion 2/3, and we obtain ${{\,\mathrm{\text {SC}}\,}}({\mathbf {z}}) = (k+1)/3$ which implies the theorem. $\square$

Clearly, the above result states the inefficiency of the best pure Nash equilibrium only when $k\ge 3$. For the remaining cases where $k\in \{1,2\}$ we present slightly more complicated instances, where the proofs rely on Lemma 5. Recall that, for 1-COF games, $\sigma (i)$ denotes the single neighbor of player *i*.

#### Theorem 8

The price of stability of 1-COF games is at least 17/15.

#### Proof

We use the following 1-COF game with six players and belief vector

$$\begin{aligned} {\mathbf {s}}= (0, 5-3\lambda , 8, 15, 18+3\lambda , 23), \end{aligned}$$

where $$\lambda \in (0,1/4)$$.

Consider the opinion vector

$$\begin{aligned} {\tilde{{\mathbf {z}}}} = (3-\lambda , 6-2\lambda , 7-6\lambda , 16+6\lambda , 17+2\lambda , 20+\lambda ). \end{aligned}$$

It can be easily seen that it has social cost ${{\,\mathrm{\text {SC}}\,}}({\tilde{{\mathbf {z}}}}, {\mathbf {s}}) = 10+12\lambda$. So, clearly, ${{\,\mathrm{\text {SC}}\,}}({\mathbf {z}}^*, {\mathbf {s}})\le 10+12\lambda$ for any optimal opinion vector ${\mathbf {z}}^*$.

Now, consider the opinion vector

$$\begin{aligned} {\mathbf {z}}= \left( \frac{5-3\lambda }{3}, \frac{10-6\lambda }{3}, \frac{31}{3}, \frac{38}{3}, \frac{59+6\lambda }{3}, \frac{64+3\lambda }{3}\right) \end{aligned}$$

with social cost ${{\,\mathrm{\text {SC}}\,}}({\mathbf {z}}, {\mathbf {s}}) = 34/3-4\lambda$. It is not hard to verify (by showing, as Lemma 1 requires, that each opinion lies in the midpoint of its player’s interval) that ${\mathbf {z}}$ is a pure Nash equilibrium; we argue that this equilibrium is unique.

We claim that, by Lemma 5, there cannot be a pure Nash equilibrium where both $\sigma (j-1) = j$ and $\sigma (j+1) = j$ for any $j\in \{2,5\}$. To see this, assume otherwise and note that the corresponding intervals satisfy the conditions of the lemma. However, by observing the belief vector ${\mathbf {s}}$, it holds that $\frac{5s_{j-1}+3s_{j+1}}{8}<s_j<\frac{3s_{j-1}+5s_{j+1}}{8}$, for $j\in \{2,5\}$, i.e., ${\mathbf {s}}$ does not satisfy the conditions of Lemma 5; this contradicts our original assumption.

The above observation, together with Lemma 2, implies that $\sigma (1) = 2$, $\sigma (3) =4$, $\sigma (4)=3$ and $\sigma (6)=5$ in any equilibrium. This leaves only $\sigma (2)\in \{1,3\}$ and $\sigma (5)\in \{4,6\}$ undefined.

Consider an equilibrium ${\mathbf {z}}'$ with $\sigma (2) = 3$; the case $\sigma (5)=4$ is symmetric. Since $\sigma (3) = 4$, Lemma 4 implies that $z'_3>s_3=8$ and, hence

7 $$\begin{aligned} z'_3-s_2>3+3\lambda .\end{aligned}$$

Since $\sigma (1) = 2$, $\sigma (2) =3$ and $z'_1 = \frac{s_1+z'_2}{2}$, Lemma 4 implies that $z'_2>s_2$ and we obtain that $z'_1>\frac{5-3\lambda }{2}$ and, hence,

8 $$\begin{aligned} s_2-z'_1<\frac{5-3\lambda }{2}.\end{aligned}$$

By inequalities (7) and (8), we get $z'_3-s_2>s_2-z'_1$, which contradicts our assumption that $\sigma (2) = 3$. So, it must hold that $\sigma (2)=1$ (and, respectively, $\sigma (5) =6$) which implies that ${\mathbf {z}}$ is the unique pure Nash equilibrium.

We conclude that the price of stability is lower-bounded by

$$\begin{aligned} \frac{{{\,\mathrm{\text {SC}}\,}}({\mathbf {z}}, {\mathbf {s}})}{{{\,\mathrm{\text {SC}}\,}}({\mathbf {z}}^*, {\mathbf {s}})}=\frac{34/3-4\lambda }{10+12\lambda }, \end{aligned}$$

and the theorem follows by taking $\lambda$ to be arbitrarily close to 0. $\square$

#### Theorem 9

The price of stability of 2-COF games is at least 8/7.

#### Proof

Consider a 2-COF game with four players *a*, *b*, *c*, and *d*, with belief vector ${\mathbf {s}}= (0, 1, 1, 2)$. Let ${\tilde{{\mathbf {z}}}} = (1,1,1,3/2)$ be an opinion vector and observe that $SC({\tilde{{\mathbf {z}}}}, {\mathbf {s}}) = 3/2$; note that ${\tilde{{\mathbf {z}}}}$ is not a pure Nash equilibrium as player *a* has an incentive to deviate. Clearly, the optimal social cost is at most 3/2.

Now consider any pure Nash equilibrium ${\mathbf {z}}$. By the structural properties of equilibria, $N_a({\mathbf {z}}, {\mathbf {s}}) = N_d({\mathbf {z}}, {\mathbf {s}}) = \{b,c\}$, while $b \in N_c({\mathbf {z}}, {\mathbf {s}})$ and $c \in N_b({\mathbf {z}}, {\mathbf {s}})$. It remains to argue about the second neighbor of *b* and *c*. We distinguish between two cases depending on whether *b* and *c* have a common second neighbor in $\{a, d\}$ or not.

In the first case, let *a* be the common neighbor; the case where *d* is that neighbor is symmetric. By Lemma 1, we have that $z_b = z_c = (1+z_a)/2$. Then, we have that $I_a({\mathbf {z}}, {\mathbf {s}}) = [0, z_b]$, $I_b({\mathbf {z}}, {\mathbf {s}}) = [z_a, 1]$, and $I_d({\mathbf {z}}, {\mathbf {s}}) = [z_b, 2]$. Note that by applying Lemma 5 on players *a*, *b*, and *d*, we obtain a contradiction to the fact that ${\mathbf {z}}$ is a pure Nash equilibrium.

In the second case, without of loss of generality, let $N_b({\mathbf {z}}, {\mathbf {s}}) = \{a, c\}$ and $N_c({\mathbf {z}}, {\mathbf {s}}) = \{b, d\}$ which, by Lemma 4, imply that $z_b \in [0, 1]$ and $z_c \in [1, 2]$. Then, Lemma 1 yields $z_a = z_c/2$, $z_b = (z_a+z_c)/2$, $z_c = (z_b+z_d)/2$, and $z_d = 1+z_b/2$. By solving this system of equations, we obtain that ${\mathbf {z}}= (4/7, 6/7, 8/7, 10/7)$ and, hence, ${{\,\mathrm{\text {SC}}\,}}({\mathbf {z}}) = 12/7$. $\square$

## Complexity of Equilibria

In this section we focus entirely on 1-COF games. We present a polynomial-time algorithm that determines whether such a game admits pure Nash equilibria, and, in case it does, allows us to compute the best and worst pure Nash equilibrium with respect to the social cost. We do so by establishing a correspondence between pure Nash equilibria and source-sink paths in a suitably defined directed acyclic graph. See Example 2 below for an instance execution of the following procedure.

Assume that we are given neighborhood information according to which each player *i* has either player $i-1$ or player $i+1$ as neighbor. From Lemma 3, such a neighborhood structure is necessary in a pure Nash equilibrium. We claim that this information is enough in order to decide whether there is a consistent opinion vector that is a pure Nash equilibrium or not. All we have to do is to use Lemma 1 and obtain *n* equations that relate the opinion of each player to her belief and her neighbor’s opinion. These equations have a unique solution which can then be verified whether it indeed satisfies the neighborhood conditions or not. So, the main idea of our algorithm is to cleverly search among all possible neighborhood structures that are not excluded by Lemma 3 for one that defines a pure Nash equilibrium.

For integers $1\le a\le b<c\le n$, let us define the *segment* *C*(*a*, *b*, *c*) to be the set of players $\{a, a+1, ..., c\}$ together with the following neighborhood information for them: $\sigma (p)=p+1$ for $p=a, ..., b$ and $\sigma (p)=p-1$ for $p=b+1, ..., c$. It can be easily seen that the neighborhood information for all players at a pure Nash equilibrium can always be decomposed into disjoint segments. Importantly, given the neighborhood information in segment *C*(*a*, *b*, *c*) and the beliefs of its players, the opinions they could have in any pure Nash equilibrium that contains this segment are uniquely defined using Lemma 1. In particular, the opinions of the players within a segment *C*(*a*, *b*, *c*) are computed as follows. First, we set $z_b = s_b+\frac{s_{b+1}-s_b}{3}$ and $z_{b+1} = s_b+\frac{2(s_{b+1}-s_b)}{3}$. Then, we set $z_p = \frac{s_p+z_{p+1}}{2}$ if $a\le p <b$, and $z_p = \frac{s_p+z_{p-1}}{2}$ if $b<p\le c$.

We remark that the opinion vector implied by a segment is not necessarily consistent to the given neighborhood structure. So, we call segment *C*(*a*, *b*, *c*) *legit* if $a\not =2$, $c\not =n-1$ (so that it can be part of a decomposition) and the uniquely defined opinions are consistent to the neighborhood information of the segment, i.e., if $|z_{\sigma (p)} - s_p| \le |z_{p'} - s_p|$ for any pair of players $p, p'$ (with $p\ne p'$) in *C*(*a*, *b*, *c*). This process appears in Algorithm 1.

A decomposition of neighborhood information for all players will consist of consecutive segments $C(a_1,b_1,c_1)$, $C(a_2,b_2,c_2)$, ..., $C(a_t,b_t,c_t)$ so that $a_1=1$, $c_t=n$, $a_\ell =c_{\ell -1}+1$ for $\ell =2, ..., t$. Such a decomposition will yield a pure Nash equilibrium if it consists of legit segments and, furthermore, the uniquely defined opinions of players in consecutive segments are consistent to the neighborhood information.

In particular, consider the directed graph *G* that has two special nodes designated as the source and the sink, and a node for each legit segment *C*(*a*, *b*, *c*). Note that *G* has ${\mathcal {O}}(n^3)$ nodes, as there are ${\mathcal {O}}(n)$ choices for each of *a*, *b*, and *c*. The source node is connected to all segment nodes *C*(1, *b*, *c*) while all segment nodes *C*(*a*, *b*, *n*) are connected to the sink. An edge from segment node *C*(*a*, *b*, *c*) to segment node $C(a',b',c')$ exists if $a'=c+1$ and the uniquely defined opinions of players in the two segments are consistent to the neighborhood information in both of them. This consistency test has to check

1. whether the leftmost opinion $z_{a'}$ in segment $C(a',b',c')$ is indeed further away from the belief $s_c$ of player *c* than the opinion $z_{c-1}$ of the designated neighbor of *c* in segment *C*(*a*, *b*, *c*), i.e., $|z_{c-1}-s_c|\le |z_{a'}-s_c|$, and whether
2. whether the rightmost opinion $z_c$ in segment *C*(*a*, *b*, *c*) is further away from the belief $s_{a'}$ of player $a'$ than the opinion $z_{a'+1}$ of the designated neighbor of $a'$ in segment $C(a',b',c')$, i.e., $|z_{a'+1}-s_{a'}|\le |z_c-s_{a'}|$.

By the definition of segments and of its edges, *G* is acyclic. This process appears in Algorithm 2.

Based on the discussion above, there is a bijection between pure Nash equilibria and source-sink paths in *G*. In addition, we can assign a weight to each node of *G* that is equal to the total cost of the players in the corresponding segment, i.e.,

$$\begin{aligned} {{\,\mathrm{weight}\,}}({C(a,b,c)}) = \sum _{a\le p \le c}{|z_p-s_p|}. \end{aligned}$$

Then, the total weight of a source-sink path ${{{\mathcal {P}}}}$ is equal to the social cost of the corresponding pure Nash equilibrium, i.e,

$$\begin{aligned} {{\,\mathrm{\text {SC}}\,}}({\mathbf {z}},{\mathbf {s}}) = \sum _{C(a,b,c) \in {{{\mathcal {P}}}}}{{{\,\mathrm{weight}\,}}({C(a,b,c)})}. \end{aligned}$$

Hence, standard algorithms for computing shortest or longest paths in directed acyclic graphs can be used not only to detect whether a pure Nash equilibrium exists, but also to compute the equilibrium of best or worst social cost.

### Theorem 10

Given a 1-COF game, deciding whether a pure Nash equilibrium exists can be done in polynomial time. Furthermore, computing a pure Nash equilibrium of highest or lowest social cost can be done in polynomial time as well.


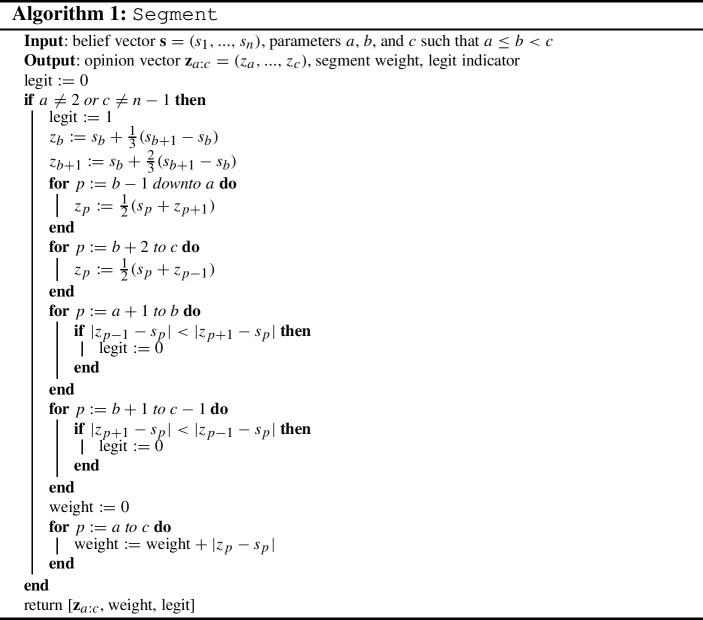
 
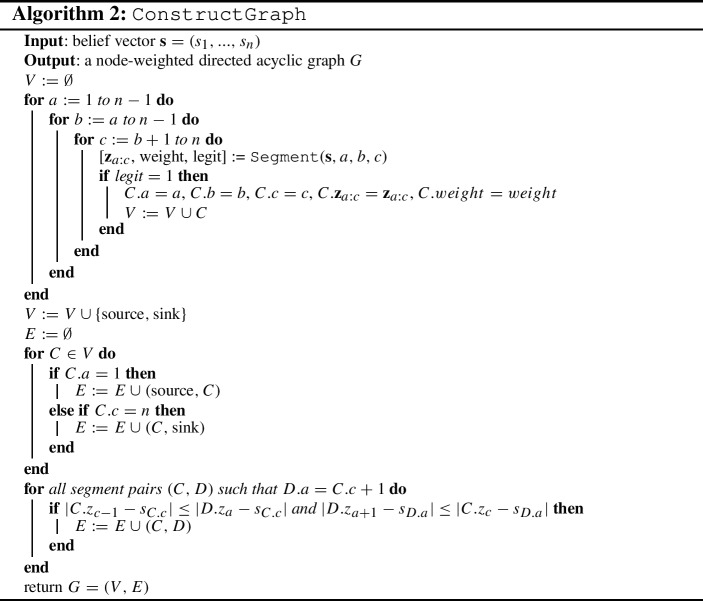


### Example 2

**Fig. 5:**
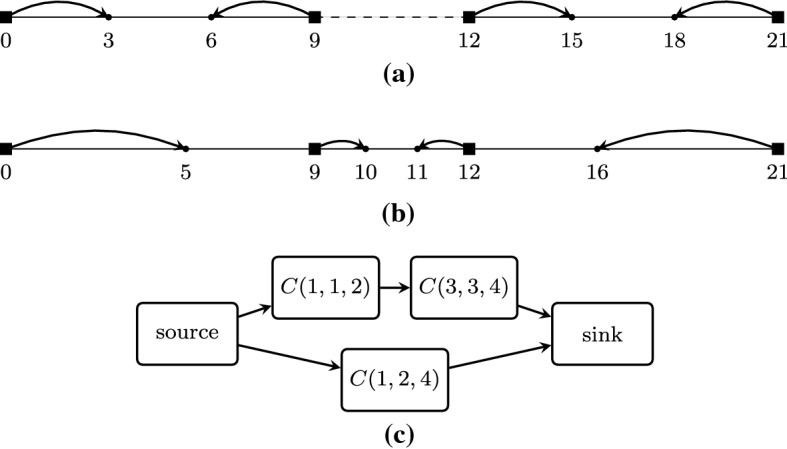
The 1-COF game considered in Example 2. **a** The legit segments *C*(1, 1, 2) and *C*(3, 3, 4) which imply the opinion vector (3, 6, 15, 18). **b** The legit segment *C*(1, 2, 4) which implies the opinion vector (5, 10, 11, 16). **c** The directed acyclic graph *G* which shows that there exist two pure Nash equilibria in the game

Consider a 1-COF game with four players with belief vector ${\mathbf {s}}= (0, 9, 12, 21)$. According to the discussion above, there are 10 segments of the form *C*(*a*, *b*, *c*) with $1\le a\le b< c\le 4$, but it can be shown that only 3 of them are legit; these are *C*(1, 1, 2), *C*(3, 3, 4) (see Fig. 5a), and *C*(1, 2, 4) (see Fig. 5b). For example, segment *C*(1, 1, 4), in which $\sigma (1)= 2$, $\sigma (2) = 1$, $\sigma (3) = 2$, and $\sigma (4) = 3$, corresponds to the opinion vector (3, 6, 9, 15). This is not consistent to the neighborhood information $\sigma (2)=1$ in the segment, as the belief of player 2 coincides with the opinion of player 3, while the opinion of player 1 is further away. The resulting directed acyclic graph *G* (see Fig. 5c) implies that there exist two pure Nash equilibria for this 1-COF game, namely the opinion vectors (3, 6, 15, 18) and $(5,10,11,16)$.       $\square$

## Upper Bounds on the Price of Anarchy

In this section we prove upper bounds on the price of anarchy of *k*-COF games. In our proof, we relate the social cost of any deterministic opinion vector, including optimal ones, to a quantity that depends only on the beliefs of the players and can be thought of as the cost of the truthful opinion vector (in which the opinion of every player is equal to her belief). In particular, we prove a lower bound on the optimal social cost (in Lemmas 11) and an upper bound on the social cost of any pure Nash equilibrium, both expressed in terms of this quantity. The bound on the price of anarchy then follows by these relations; see the proof of Theorem 12.

Consider an *n*-player *k*-COF game with belief vector ${\mathbf {s}}=(s_1, ..., s_n)$. For player *i*, we denote by $\ell ^*(i)$ and $r^*(i)$ the integers in [*n*] such that $\ell ^*(i)\le i\le r^*(i)$, $r^*(i)-\ell ^*(i) = k$, and $|s_{r^*(i)}-s_{\ell ^*(i)}|$ is minimized. The proof of the next lemma exploits linear programming and duality.

### Lemma 11

Consider a *k*-COF game with belief vector ${\mathbf {s}}= (s_1, ..., s_n)$ and let ${\mathbf {z}}$ be any deterministic opinion vector. Then,

$$\begin{aligned} {{\,\mathrm{\text {SC}}\,}}({\mathbf {z}},{\mathbf {s}}) \ge \frac{1}{2(k+1)}\sum _{i=1}^n{|s_{r^*(i)}-s_{\ell ^*(i)}|}. \end{aligned}$$

### Proof

Consider any deterministic opinion vector ${\mathbf {z}}$ and let $\pi$ be a permutation of the players so that $z_{\pi (j)}\le z_{\pi (j+1)}$ for each $j\in [n-1]$. We refer to player $\pi (j)$ as the player with rank *j*.^1^ For each player *i*, we will identify an effective neighborhood $F_i({\mathbf {z}},{\mathbf {s}})$ that consists of $k+1$ players with consecutive ranks and includes player *i*. Define ${\tilde{\ell }}(i)$ and ${\tilde{r}}(i)$ to be the players in $F_i({\mathbf {z}},{\mathbf {s}})$ with the lowest and highest belief, respectively. In the extreme case where all players in $F_i({\mathbf {z}},{\mathbf {s}})$ have the same belief, we let ${\tilde{\ell }}(i)$ and ${\tilde{r}}(i)$ be the players with the lowest and highest ranks, respectively. The effective neighborhood will be defined in such a way that it satisfies the properties ${{\,\mathrm{cost}\,}}_i({\mathbf {z}},{\mathbf {s}})\ge z_{{\tilde{r}}(i)}-z_i$ and ${{\,\mathrm{cost}\,}}_i({\mathbf {z}},{\mathbf {s}})\ge z_i-z_{{\tilde{\ell }}(i)}$.

**Fig. 6:**
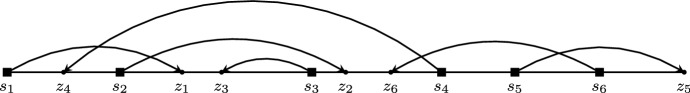
An example of the quantities used in the proof of Lemma 11. Let $k=2$ and $i=4$. Then, the neighborhood of player 4 is $N_4({\mathbf {z}},{\mathbf {s}}) = \{2,6\}$, the smallest contiguous interval containing the opinions of players in $N_4({\mathbf {z}},{\mathbf {s}})\cup \{4\}$ is $J_4({\mathbf {z}},{\mathbf {s}}) = [z_4,z_6]$, the set of players with opinions in $J_4({\mathbf {z}},{\mathbf {s}})$ is $D_4({\mathbf {z}},{\mathbf {s}}) = \{1,2,3,4,6\}$, the effective neighborhood is $F_4({\mathbf {z}},{\mathbf {s}}) = \{1,3,4\}$, and, hence, ${\tilde{\ell }}(4)=1$, and ${\tilde{r}}(4)=4$

Let $N_i({\mathbf {z}},{\mathbf {s}})$ denote the neighborhood of player *i*, i.e., the set of players (not including *i*) with the *k* closest opinions to the belief $s_i$ of player *i*. Let $J_i({\mathbf {z}},{\mathbf {s}})$ be the smallest contiguous interval containing all opinions of players in $N_i({\mathbf {z}},{\mathbf {s}})\cup \{i\}$ and let $D_i({\mathbf {z}},{\mathbf {s}})$ be the set of players with opinions in $J_i({\mathbf {z}},{\mathbf {s}})$. Clearly, $|D_i({\mathbf {z}},{\mathbf {s}})|\ge k+1$. We define $F_i({\mathbf {z}},{\mathbf {s}})$ to be a subset of $D_i({\mathbf {z}},{\mathbf {s}})$ that consists of $k+1$ players with consecutive ranks including player *i*. See Fig. 6 for an illustrative example of all quantities defined above.

Let $\ell '(i)$ and $r'(i)$ be the players in $N_i({\mathbf {z}},{\mathbf {s}})$ with the leftmost and rightmost opinion. In order to show that the definition of $F_i({\mathbf {z}},{\mathbf {s}})$ satisfies the two desired properties, we distinguish between three different cases depending on the location of opinion $z_i$ among the players in $N_i({\mathbf {z}},{\mathbf {s}})\cup \{i\}$.

- **Case I:** Player *i* has neither the leftmost nor the rightmost opinion in $N_i({\mathbf {z}},{\mathbf {s}})\cup \{i\}$, i.e., $z_{\ell '(i)}<z_i<z_{r'(i)}$.^2^ In this case, $J_i({\mathbf {z}},{\mathbf {s}})=[z_{\ell '(i)},z_{r'(i)}]$. Then, the definition of $N_i({\mathbf {z}},{\mathbf {s}})$ implies that ${{\,\mathrm{cost}\,}}_i({\mathbf {z}},{\mathbf {s}})\ge z_{r'(i)}-z_i$ and ${{\,\mathrm{cost}\,}}_i({\mathbf {z}},{\mathbf {s}})\ge z_i-z_{\ell '(i)}$. Hence, ${{\,\mathrm{cost}\,}}_i({\mathbf {z}},{\mathbf {s}})\ge |z_j-z_i|$ for every $z_j\in J_i({\mathbf {z}},{\mathbf {s}})$ or, equivalently, $j\in D_i({\mathbf {z}},{\mathbf {s}})$ and, subsequently, for each $j\in F_i({\mathbf {z}},{\mathbf {s}})$. This implies the two desired properties ${{\,\mathrm{cost}\,}}_i({\mathbf {z}},{\mathbf {s}})\ge z_{{\tilde{r}}(i)}-z_i$ and ${{\,\mathrm{cost}\,}}_i({\mathbf {z}},{\mathbf {s}})\ge z_i - z_{{\tilde{\ell }}(i)}$.
- **Case II:** Player *i* has the leftmost opinion in $N_i({\mathbf {z}},{\mathbf {s}})\cup \{i\}$, i.e., $z_i\le z_{\ell '(i)}$. Then, $J_i({\mathbf {z}},{\mathbf {s}})=[z_i,z_{r'(i)}]$. Now, the definition of $N_i({\mathbf {z}},{\mathbf {s}})$ implies that ${{\,\mathrm{cost}\,}}_i({\mathbf {z}},{\mathbf {s}})\ge z_{r'(i)}-z_i$ and, hence, ${{\,\mathrm{cost}\,}}_i({\mathbf {z}},{\mathbf {s}})\ge |z_j-z_i|$ for every $z_j\in J_i({\mathbf {z}},{\mathbf {s}})$ or, equivalently, $j\in D_i({\mathbf {z}},{\mathbf {s}})$ and, subsequently, for each $j\in F_i({\mathbf {z}},{\mathbf {s}})$. Again, this implies the two desired properties.
- **Case III:** Player *i* has the rightmost opinion in $N_i({\mathbf {z}},{\mathbf {s}})\cup \{i\}$, i.e., $z_i\ge z_{r'(i)}$. Then, $J_i({\mathbf {z}},{\mathbf {s}})=[z_{\ell '(i)},z_i]$. Now, the definition of $N_i({\mathbf {z}},{\mathbf {s}})$ implies that ${{\,\mathrm{cost}\,}}_i({\mathbf {z}},{\mathbf {s}})\ge z_i-z_{\ell '(i)}$ and, hence, ${{\,\mathrm{cost}\,}}_i({\mathbf {z}},{\mathbf {s}})\ge |z_j-z_i|$ for every $z_j\in J_i({\mathbf {z}},{\mathbf {s}})$ or, equivalently, $j\in D_i({\mathbf {z}},{\mathbf {s}})$ and, subsequently, for every $j\in F_i({\mathbf {z}},{\mathbf {s}})$. Again, the two desired properties follow.

By setting the variable $t_i$ equal to ${{\,\mathrm{cost}\,}}_i({\mathbf {z}},{\mathbf {s}})$ for $i\in [n]$, the discussion above and the fact that ${{\,\mathrm{cost}\,}}_i({\mathbf {z}},{\mathbf {s}})\ge |s_i-z_i|$ imply that the opinion vector ${\mathbf {z}}$ together with ${\mathbf {t}}=(t_1, \dots , t_n)$ is a feasible solution to the following linear program:

$$\begin{aligned} \text{ minimize }&\quad \sum _{i\in [n]}{t_i}\\ \text{ subject } \text{ to }&\quad t_i+z_i\ge s_i, \forall i\in [n]\\&\quad t_i-z_i\ge -s_i, \forall i\in [n]\\&\quad t_i+z_i-z_{{\tilde{r}}(i)} \ge 0, \forall i \in [n] \text{ such } \text{ that } {\tilde{r}}(i)\not =i\\&\quad t_i+z_{{\tilde{\ell }}(i)}-z_i \ge 0, \forall i \in [n] \text{ such } \text{ that } {\tilde{\ell }}(i)\not =i\\&\quad t_i,z_i\ge 0, \forall i\in [n] \end{aligned}$$

Using the dual variables $\alpha _i$, $\beta _i$, $\gamma _i$, and $\delta _i$ associated with the four constraints of the above LP, we obtain its dual LP:

$$\begin{aligned} \text{ maximize }&\quad \sum _{i\in [n]}{s_i \alpha _i} - \sum _{i\in [n]}{s_i \beta _i}\\ \text{ subject } \text{ to }&\quad \alpha _i+\beta _i+\gamma _i \cdot \mathbb {1}\{{\tilde{r}}(i)\not =i\} +\delta _i \cdot \mathbb {1}\{{\tilde{\ell }}(i)\not =i\} \le 1, \forall i\in [n]\\&\quad \alpha _i-\beta _i+\gamma _i\cdot \mathbb {1}\{{\tilde{r}}(i)\not =i\}-\delta (i)\cdot \mathbb {1}\{{\tilde{\ell }}(i)\not =i\}-\sum _{j\not =i:{\tilde{r}}(j)=i}{\gamma _j} \\&\quad +\sum _{j\not =i:{\tilde{\ell }}(j)=i}{\delta _j}\le 0, \forall i\in [n]\\&\quad \alpha _i, \beta _i, \gamma _i, \delta _i \ge 0 \end{aligned}$$

The indicator $\mathbb {1}\{X\}$ is equal to 1 when the condition *X* is true, and 0 otherwise. We will show that the solution defined as

$$\begin{aligned} \alpha _i=\frac{|\{j\in [n]:{\tilde{r}}(j)=i\}|}{2(k+1)}, \\ \beta _i=\frac{|\{j\in [n]:{\tilde{\ell }}(j)=i\}|}{2(k+1)}, \\ \gamma _i=\delta _i=\frac{1}{2(k+1)}, \end{aligned}$$

is a feasible dual solution. Indeed, to see why the first dual constraint is satisfied, first observe that player *i* belongs to at most $2k+1$ different effective neighborhoods. Hence, player *i* can have the lowest or highest belief among the players in the effective neighborhood of at most $2k+1$ players (implying that $\alpha _i+\beta _i\le 1-\frac{1}{2(k+1)}$) when ${\tilde{r}}(i)=i$ or ${\tilde{\ell }}(i)=i$ and of at most 2*k* players (implying that $\alpha _i+\beta _i\le 1-\frac{1}{k+1}$) when ${\tilde{r}}(i)\not =i$ and ${\tilde{\ell }}(i)\not =i$. The first constraint follows.

It remains to show that the second constraint is satisfied as well (with equality). We do so by distinguishing between three cases:

- When ${\tilde{r}}(i)\not =i$ and ${\tilde{\ell }}(i)\not =i$, the dual solution guarantees that $\alpha _i=\sum _{j\not =i:{\tilde{r}}(j)=i}{\gamma _j}$ and the term $\alpha _i$ in the left-hand side of the second constraint cancels out with the sum of $\gamma$’s. Similarly, $\beta _i=\sum _{j\not =i:{\tilde{\ell }}(j)=i}{\delta _j}$ and the term $\beta _i$ cancels out with the sum of $\delta$’s. Also, the terms $\gamma _i$ and $\delta _i$ are both equal to $\frac{1}{2(k+1)}$ and cancel out as well.
- When ${\tilde{r}}(i)=i$ (then, clearly, ${\tilde{\ell }}(i)\not =i$), we have that $\alpha _i=\delta _i\cdot \mathbb {1}\{{\tilde{\ell }}(i)\not =i\} +\sum _{j\not =i:{\tilde{r}}(j)=i}{\gamma _j}$ (canceling out the first, fourth and fifth terms) and $\beta _i=\sum _{j\not =i:{\tilde{\ell }}(j)=i}{\delta _j}$ (canceling out the second and sixth terms), and the second constraint is satisfied with equality as the third term is zero.
- Finally, when ${\tilde{\ell }}(i)=i$ (now, it is ${\tilde{r}}(i)\not =i$), we have that $\alpha _i=\sum _{j\not =i:{\tilde{r}}(j)=i}{\gamma _j}$ (canceling out the first and fifth terms) and $\beta _i=\gamma _i\cdot \mathbb {1}\{{\tilde{r}}(i)\not =i\}+\sum _{j\not =i:{\tilde{\ell }}(j)=i}{\delta _j}$ (canceling out the second, third and sixth terms), and the second constraint is satisfied with equality as the fourth term is zero.

So, the social cost of the solution ${\mathbf {z}}$ is lower-bounded by the objective value of the primal LP which, by duality, is lower-bounded by the objective value of the dual LP. Hence

$$\begin{aligned} {{\,\mathrm{\text {SC}}\,}}({\mathbf {z}},{\mathbf {s}})&\ge \sum _{i\in [n]}{s_i \alpha _i} - \sum _{i\in [n]}{s_i \beta _i}\\&= \frac{1}{2(k+1)} \left( \sum _{i\in [n]}{|\{j\in [n]:{\tilde{r}}(j)=i\}|s_i} - \sum _{i\in [n]}{|\{j\in [n]:{\tilde{\ell }}(j)=i\}|s_i}\right) \\&= \frac{1}{2(k+1)} \sum _{i\in [n]}{(s_{{\tilde{r}}(i)}-s_{{\tilde{\ell }}(i)})}\\&= \frac{1}{2(k+1)} \sum _{i\in [n]}{|s_{{\tilde{r}}(i)}-s_{{\tilde{\ell }}(i)}|}. \end{aligned}$$

The last equality follows since $s_{{\tilde{r}}(i)}\ge s_{{\tilde{\ell }}(i)}$, by the definition of ${\tilde{r}}(i)$ and ${\tilde{\ell }}(i)$.

Note that for each player *i*, there are at least $k+1$ beliefs of different players with values in $[s_{{\tilde{\ell }}(i)}, s_{{\tilde{r}}(i)}]$, including player *i*. By the definition of $\ell ^*(i)$ and $r^*(i)$ for each player *i*, the above inequality yields

$$\begin{aligned} {{\,\mathrm{\text {SC}}\,}}({\mathbf {z}},{\mathbf {s}})&\ge \frac{1}{2(k+1)} \sum _{i\in [n]}{|s_{r^*(i)}-s_{\ell ^*(i)}|}, \end{aligned}$$

as desired. $$\square $$

We are now ready to prove our upper bound on the price of anarchy for *k*-COF games. In our proof, we exploit the monotonicity of opinions in a pure Nash equilibrium and we associate the cost of each player in the equilibrium to the same quantity used in the statement of Lemma 11.

### Theorem 12

The price of anarchy of *k*-COF games over pure Nash equilibria is at most $4(k+1)$.

### Proof

Consider a *k*-COF game with belief vector ${\mathbf {s}}=(s_1, \dots , s_n)$, and let ${\mathbf {z}}^*=(z_1^*, \dots , z_n^*)$ be any opinion vector that minimizes the social cost. By Lemma 11, we have

9 $$\begin{aligned} {{\,\mathrm{\text {SC}}\,}}({\mathbf {z}}^*,{\mathbf {s}})&\ge \frac{1}{2(k+1)}\sum _{i=1}^n{|s_{r^*(i)}-s_{\ell ^*(i)}|}. \end{aligned}$$

Now, consider any pure Nash equilibrium ${\mathbf {z}}$ of the game. We will show that

10 $$\begin{aligned} {{\,\mathrm{\text {SC}}\,}}({\mathbf {z}},{\mathbf {s}})&\le 2\sum _{i=1}^n{|s_{r^*(i)}-s_{\ell ^*(i)}|}, \end{aligned}$$

and the theorem will then follow by inequalities (9) and (10).

The rest of this proof is, therefore, devoted to showing inequality (10). To this end, we will show that, for any player *i*, we have ${{\,\mathrm{cost}\,}}_i({\mathbf {z}},{\mathbf {s}}) \le 2(s_{r^*(i)}-s_{\ell ^*(i)})$. Then, inequality (10) will follow by summing over all players.

Consider an arbitrary player *i* and, without loss of generality, let us assume that $z_i\ge s_i$ (the case $z_i\le s_i$ is symmetric). Recall that $\ell (i)$ and *r*(*i*) denote the players in $N_i({\mathbf {z}},{\mathbf {s}})\cup \{i\}$ with the leftmost and rightmost point, respectively, in $I_i({\mathbf {z}},{\mathbf {s}})$ and note that $r(i) - \ell (i) = k$. First, observe that if $z_{r(i)}=z_i$, the assumption $z_i\ge s_i$ implies that all players in $N_i({\mathbf {z}},{\mathbf {s}})\cup \{i\}$ have opinions at $s_i$ (since, by Lemma 1, $z_i$ is in the midpoint of interval $I_i({\mathbf {z}},{\mathbf {s}})$ at equilibrium). In this case, ${{\,\mathrm{cost}\,}}_i({\mathbf {z}},{\mathbf {s}})=0$ and the desired inequality holds trivially. So, in the following, we assume that $r(i)>i$ and $z_{r(i)}>z_i$, i.e., $z_{r(i)}$ is at the right of $z_i$ which in turn is at the right of (or coincides with) $s_i$.

Recall that, for player *i*, $\ell ^*(i)$ and $r^*(i)$ denote the integers in [*n*] such that $\ell ^*(i)\le i\le r^*(i)$, $r^*(i)-\ell ^*(i) = k$, and $|s_{r^*(i)}-s_{\ell ^*(i)}|$ is minimized. Since $r(i) - \ell (i) = r^*(i) - \ell ^*(i) = k$, we distinguish between two main cases depending on the relative order of *r*(*i*) and $r^*(i)$.

**Fig. 7:**
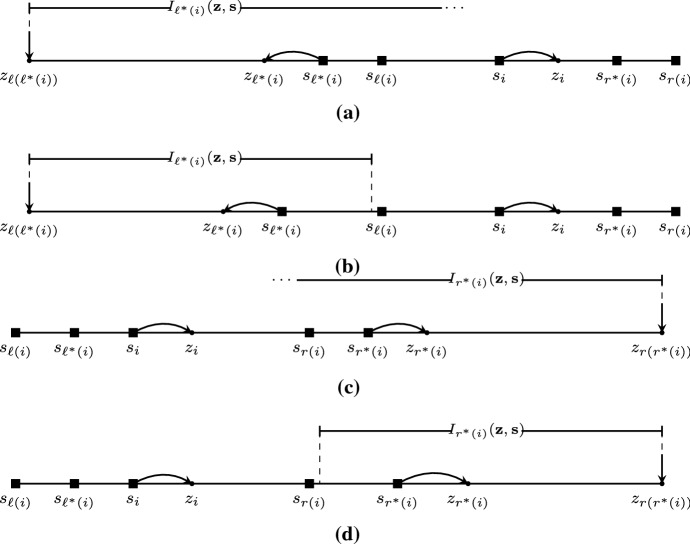
Indicative examples of the different cases in the proof of Theorem 12. Subfigures **a** and **b** concern Case I, as $r(i)>r^*(i)$ and $\ell (i)>\ell ^*(i)$, while subfigures **c** and **d** fall under Case II, as $r(i)\le r^*(i)$ and $\ell (i)\le \ell ^*(i)$

- **Case I:** $r(i)>r^*(i)$ and $\ell (i)>\ell ^*(i)$. Since $z_{r(i)}$ is at the right of $s_i$ and $\ell ^*(i)$ does not belong to the neighborhood of player *i* (while player *r*(*i*) does so by definition), $z_{\ell ^*(i)}$ is at the left of $s_i$ and, furthermore, $z_{r(i)}-s_i\le s_i-z_{\ell ^*(i)}$ or, equivalently,
  11 $$\begin{aligned} z_{r(i)}&\le 2s_i-z_{\ell ^*(i)}. \end{aligned}$$
  This yields
  12 $$\begin{aligned} {{\,\mathrm{cost}\,}}_i({\mathbf {z}},{\mathbf {s}})&= z_{r(i)}-z_i \le 2s_i-z_{\ell ^*(i)}-z_i. \end{aligned}$$
  These inequalities will be useful in several places of the proof for this case below. If $z_{\ell ^*(i)}\ge s_{\ell ^*(i)}$ then, since $r^*(i)\ge i$ and $z_i\ge s_i$, inequality (12) becomes ${{\,\mathrm{cost}\,}}_i({\mathbf {z}},{\mathbf {s}})\le s_i-s_{\ell ^*(i)} \le s_{r^*(i)}-s_{\ell ^*(i)}$ and the desired inequality follows. So, in the following, we assume that $z_{\ell ^*(i)}<s_{\ell ^*(i)}$ i.e., $z_{\ell ^*(i)}$ is (strictly) at the left of $s_{\ell ^*(i)}$. Hence, $\ell ^*(i)$ has her leftmost neighbor with $z_{\ell (\ell ^*(i))}<z_{\ell ^*(i)}$ and, by Lemma 1,
  13 $$\begin{aligned} z_{\ell ^*(i)}&= \frac{z_{\ell (\ell ^*(i))}+\max \{s_{\ell ^*(i)},z_{r(\ell ^*(i))}\}}{2}. \end{aligned}$$
  Since $r^*(i)-\ell ^*(i)=k$ and $\ell (\ell ^*(i))<\ell ^*(i)$, we have $r^*(i) - \ell (\ell ^*(i)) > k$, and, therefore, $r^*(i)$ does not belong to the neighborhood of $\ell ^*(i)$. Hence, $s_{\ell ^*(i)}-z_{\ell (\ell ^*(i))}\le z_{r^*(i)}-s_{\ell ^*(i)}$ or, equivalently
  14 $$\begin{aligned} z_{\ell (\ell ^*(i))}&\ge 2s_{\ell ^*(i)}-z_{r^*(i)} \ge 2s_{\ell ^*(i)}-2s_i+z_{\ell ^*(i)}, \end{aligned}$$
  where the second inequality follows by our case assumption $z_{r^*(i)}\le z_{r(i)}$ and inequality (11). We now further distinguish between two cases, depending on whether player *i* belongs to the neighborhood of player $\ell ^*(i)$ or not.
  - **Case I.1:** $i\in N_{\ell ^*(i)}({\mathbf {z}},{\mathbf {s}})$; see also Fig. 7a for an example of this case. Then, we have $z_i\le z_{r(\ell ^*(i))}$ and, subsequently,
    15 $$\begin{aligned} \max \{s_{\ell ^*(i)},z_{r(\ell ^*(i))}\}&\ge z_{r(\ell ^*(i))} \ge z_i. \end{aligned}$$
    Using inequalities (14) and (15), (13) yields
    $$\begin{aligned} z_{\ell ^*(i)}&\ge s_{\ell ^*(i)}-s_i+\frac{z_{\ell ^*(i)}}{2}+\frac{z_i}{2}, \end{aligned}$$
    which implies that $z_{\ell ^*(i)} \ge 2s_{\ell ^*(i)}-2s_i+z_i$. Now, inequality (12) becomes
    $$\begin{aligned} {{\,\mathrm{cost}\,}}_i({\mathbf {z}},{\mathbf {s}})&\le 4s_i-2s_{\ell ^*(i)}-2z_i \le 2s_i-2s_{\ell ^*(i)} \le 2(s_{r^*(i)}-s_{\ell ^*(i)}) \end{aligned}$$
    as desired. The second inequality follows since $z_i\ge s_i$ and the last one follows since $r^*(i)\ge i$.
  - **Case I.2:** $i\not \in N_{\ell ^*(i)}({\mathbf {z}},{\mathbf {s}})$; see also Fig. 7b for an example. Then, we have $s_{\ell ^*(i)}-z_{\ell (\ell ^*(i))} \le z_i-s_{\ell ^*(i)}$, which implies that $z_{\ell (\ell ^*(i))} \ge 2s_{\ell ^*(i)}-z_i$. Using this inequality together with the fact that $\max \{s_{\ell ^*(i)},z_{r(\ell ^*(i))}\}\ge s_{\ell ^*(i)}$, (13) yields
    $$\begin{aligned} z_{\ell ^*(i)}&\ge \frac{3s_{\ell ^*(i)}-z_i}{2} \end{aligned}$$
    and inequality (12) becomes
    $$\begin{aligned} {{\,\mathrm{cost}\,}}_i({\mathbf {z}},{\mathbf {s}})&\le 2s_i-\frac{3}{2}s_{\ell ^*(i)}-\frac{z_i}{2} \le \frac{3}{2}s_i-\frac{3}{2}s_{\ell ^*(i)} \le 2(s_{r^*(i)}-s_{\ell ^*(i)}), \end{aligned}$$
    as desired. The second last inequality follows since $z_i\ge s_i$ and the last one follows since $r^*(i)\ge i$.
- **Case II:** $r(i)\le r^*(i)$ and $\ell (i)\le \ell ^*(i)$. Since $z_i$ is in the midpoint of the interval $I_i({\mathbf {z}},{\mathbf {s}})$ and $z_{r(i)}$ is the rightmost opinion in $I_i({\mathbf {z}},{\mathbf {s}})$, we have
  $$\begin{aligned} z_i = \frac{\min \{s_i,z_{\ell (i)}\} + z_{r(i)}}{2} \le \frac{z_{\ell (i)}+z_{r(i)}}{2}\le \frac{z_{\ell ^*(i)}+z_{r^*(i)}}{2}. \end{aligned}$$
  Since $s_i\le z_i$, the last inequality yields
  16 $$\begin{aligned} z_{\ell ^*(i)}&\ge 2s_i-z_{r^*(i)}. \end{aligned}$$
  We also have
  17 $$\begin{aligned} {{\,\mathrm{cost}\,}}_i({\mathbf {z}},{\mathbf {s}})&= z_{r(i)}-z_i \le z_{r^*(i)}-z_i. \end{aligned}$$
  If $z_{r^*(i)}\le s_{r^*(i)}$ then, since $s_{\ell ^*(i)} \le s_i\le z_i$, inequality (17) yields ${{\,\mathrm{cost}\,}}_i({\mathbf {z}},{\mathbf {s}})\le s_{r^*(i)}-s_i\le s_{r^*(i)}-s_{\ell ^*(i)}$, which is even stronger than the desired inequality. So, in the following we assume that $z_{r^*(i)}>s_{r^*(i)}$, i.e., $z_{r^*(i)}$ is at the right of $s_{r^*(i)}$. Since $z_{r^*(i)}$ is in the midpoint of the interval $I_{r^*(i)}({\mathbf {z}},{\mathbf {s}})$, we have that $r(r^*(i)) > r^*(i)$ and, therefore,
  18 $$\begin{aligned} z_{r^*(i)}&= \frac{\min \{s_{r^*(i)},z_{\ell (r^*(i))}\}+z_{r(r^*(i))}}{2}. \end{aligned}$$
  Moreover, since $r(r^*(i)) - \ell ^*(i)> r^*(i) - \ell ^*(i) = k$, player $\ell ^*(i)$ does not belong to the neighborhood of player $r^*(i)$. Hence, $z_{r(r^*(i))}-s_{r^*(i)}\le s_{r^*(i)}-z_{\ell ^*(i)}$ which, together with inequality (16), yields that
  19 $$\begin{aligned} z_{r(r^*(i))}&\le 2s_{r^*(i)}-z_{\ell ^*(i)} \le 2s_{r^*(i)}-2s_i+z_{r^*(i)}. \end{aligned}$$
  We now further distinguish between two cases, depending on whether player *i* belongs to the neighborhood of player $r^*(i)$ or not.
  - **Case II.1:** $i\in N_{r^*(i)}({\mathbf {z}},{\mathbf {s}})$; see also Fig. 7c for an example. Then, using the fact that $\min \{s_{r^*(i)},z_{\ell (r^*(i))}\} \le z_{\ell (r^*(i))} \le z_i$ and inequality (19), equation (18) becomes
    $$\begin{aligned} z_{r^*(i)} \le \frac{z_i+2s_{r^*(i)}-2s_i+z_{r^*(i)}}{2} \end{aligned}$$
    and, equivalently, $z_{r^*(i)} \le z_i+2s_{r^*(i)}-2s_i$. Hence, inequality (17) yields
    $$\begin{aligned} {{\,\mathrm{cost}\,}}_i({\mathbf {z}},{\mathbf {s}})&\le 2s_{r^*(i)}-2s_i \le 2(s_{r^*(i)}-s_{\ell ^*(i)}), \end{aligned}$$
    as desired. The last inequality follows since $\ell ^*(i)\le i$.
  - **Case II.2:** $i\not \in N_{r^*(i)}({\mathbf {z}},{\mathbf {s}})$; see Fig. 7d for an example. Since *i* does not belong to the neighborhood of player $r^*(i)$ but player $r(r^*(i))$ does, we have that $z_{r(r^*(i))}-s_{r^*(i)} \le s_{r^*(i)}-z_i$ or, equivalently, $z_{r(r^*(i))} \le 2s_{r^*(i)}-z_i$. Then, using also the fact that $\min \{s_{r^*(i)},z_{\ell (r^*(i))}\} \le s_{r^*(i)}$, equation (18) becomes
    $$\begin{aligned} z_{r^*(i)}\le \frac{3s_{r^*(i)}-z_i}{2} \end{aligned}$$
    and (17) yields
    $$\begin{aligned} {{\,\mathrm{cost}\,}}_i({\mathbf {z}},{\mathbf {s}})&\le \frac{3}{2}(s_{r^*(i)}-z_i) \le \frac{3}{2}(s_{r^*(i)}-s_{\ell ^*(i)}), \end{aligned}$$
    which is even stronger than the desired inequality. The last inequality follows since $z_i\ge s_i$ and $\ell ^*(i)\le i$.

So, we have shown that in the pure Nash equilibrium ${\mathbf {z}}$ and for any player *i*, we have that ${{\,\mathrm{cost}\,}}_i({\mathbf {z}},{\mathbf {s}}) \le 2(s_{r^*(i)}-s_{\ell ^*(i)})$. By summing over all players, we obtain inequality (10) and the theorem follows. $\square$

## An Improved Bound on the Price of Anarchy for 1-COF Games

For the case of 1-COF games we can prove an even stronger statement following a similar proof roadmap as in the previous section, but using simpler (and shorter) arguments. We denote by $\eta (i)$ the player (other than *i*) that minimizes the distance $|s_i-s_{\eta (i)}|$; note that $\eta (i) \in \{i-1,i+1\}$. The proof of the next lemma (which can be thought of as a stronger version of Lemma 11 for 1-COF games) relies on a particular charging scheme that allows us to lower-bound the cost of each player in any deterministic opinion vector.

### Lemma 13

Consider a 1-COF game with belief vector ${\mathbf {s}}= (s_1, \dots , s_n)$ and let ${\mathbf {z}}$ be any deterministic opinion vector. Then,

$$\begin{aligned} {{\,\mathrm{\text {SC}}\,}}({\mathbf {z}},{\mathbf {s}}) \ge \frac{1}{3}\sum _{i=1}^n{|s_i-s_{\eta (i)}|}. \end{aligned}$$

### Proof

We begin by classifying the players into groups and, subsequently, we show how the costs of different groups can be combined so that the lemma holds. We call a player *i* with $z_i\notin [s_{i-1}, s_{i+1}]$ a *kangaroo* player and associate the quantity ${{\,\mathrm{excess}\,}}_i$ with her. If $z_i \in [s_j, s_{j+1}]$ for some $j>i$, we say that the players in the set $C_i=\{i+1, ..., j\}$ are *covered* by player *i* and define ${{\,\mathrm{excess}\,}}_i=z_i-s_j$. Otherwise, if $z_i \in [s_{j-1}, s_j]$ for some $j<i$, we say that the players in the set $C_i=\{j, ..., i-1\}$ are covered by player *i* and define ${{\,\mathrm{excess}\,}}_i=s_j-z_i$.

**Fig. 8:**
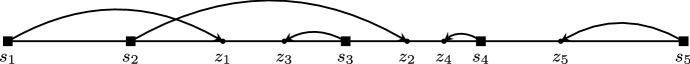
An example with kangaroos, covered, large, and small players. In particular, $1 \in {{{\mathcal {K}}}}$ as $z_1\notin [s_1,s_2]$, $2 \in {{{\mathcal {K}}}} \cap {{{\mathcal {C}}}}$ as she is covered by player 1 and, in addition, $z_2 \notin [s_1, s_3]$. Similarly, $3 \in {{{\mathcal {C}}}}$ as she is covered by player 2, while 4 and 5 are neither kangaroo nor covered. Since ${{\,\mathrm{cost}\,}}_4({\mathbf {z}},{\mathbf {s}})< \frac{1}{3}(s_4-s_3)$, it is $4 \in S$, while, since ${{\,\mathrm{cost}\,}}_5({\mathbf {z}},{\mathbf {s}}) \ge \frac{1}{3}(s_5-s_4)$, we have $5 \in L$

Let ${{{\mathcal {K}}}}$ be the set of kangaroo players and ${{{\mathcal {C}}}}$ the set of players that are covered by a kangaroo; these need not be disjoint. We now partition the players not in ${{{\mathcal {K}}}} \cup {{{\mathcal {C}}}}$ into the set *L* of *large* players such that, for any $i \in L$, it holds ${{\,\mathrm{cost}\,}}_i({\mathbf {z}},{\mathbf {s}})\ge \frac{1}{3}(|s_i-s_{\eta (i)}|)$, and the set *S* that contains the remaining players who we call *small*. See also Fig. 8 for an example of these sets.

We proceed to prove five useful properties (Claims 13.1–13.5); recall that $\sigma (i)$ denotes the single neighbor of player *i*.

### Claim 13.1

Let $$i\in {{{\mathcal {K}}}}$$. Then, $${{\,\mathrm{cost}\,}}_i({\mathbf {z}},{\mathbf {s}})-{{\,\mathrm{excess}\,}}_i \ge \frac{1}{3}(|s_i-s_{\eta (i)}|+\sum _{j\in C_i}{|s_j-s_{\eta (j)}|})$$.

### Proof

We assume that $z_i>s_i$ (the other case is symmetric). Let $\ell$ be the player with the rightmost belief that is covered by *i*. Then, ${{\,\mathrm{excess}\,}}_i=z_i-s_{\ell }$. We have

$$\begin{aligned} {{\,\mathrm{cost}\,}}_i({\mathbf {z}},{\mathbf {s}})-{{\,\mathrm{excess}\,}}_i&= \max \{|s_i-z_i|,|z_i-z_{\sigma (i)}|\}-(z_i-s_\ell )\\&\ge s_\ell -s_i=\sum _{j=i}^{\ell -1}(s_{j+1}-s_j)\\ {}&\ge \frac{1}{3}(|s_i-s_{\eta (i)}|+\sum _{j\in C_i}{|s_j-s_{\eta (j)}|})\end{aligned}$$

as desired. $$\square $$

### Claim 13.2

Let $i\in S$ such that $\sigma (i)\in {{{\mathcal {K}}}}$. Then, ${{\,\mathrm{cost}\,}}_i({\mathbf {z}},{\mathbf {s}})+{{\,\mathrm{excess}\,}}_{\sigma (i)} \ge \frac{1}{3}|s_i-s_{\eta (i)}|$.

### Proof

We assume that $\sigma (i)>i$ (the other case is symmetric). If $z_{\sigma (i)}>s_{\sigma (i)}$, then

$$\begin{aligned} {{\,\mathrm{cost}\,}}_i({\mathbf {z}},{\mathbf {s}})&= \max \{|s_i-z_i|,|z_i-z_{\sigma (i)}|\}\\&\ge \frac{1}{2}(z_{\sigma (i)}-s_i) > \frac{1}{2}(s_{\sigma (i)}-s_i)\\&\ge \frac{1}{3}|s_i-s_{\eta (i)}|, \end{aligned}$$

which contradicts the fact that *i* is a small player. Hence, $z_{\sigma (i)}\in [s_i,s_{\sigma (i)}]$, otherwise player *i* would be covered. Let *j* be the player with the leftmost belief that is covered by player $\sigma (i)$. Then, ${{\,\mathrm{excess}\,}}_{\sigma (i)}=s_j-z_{\sigma (i)}$. We have

$$\begin{aligned} {{\,\mathrm{cost}\,}}_i({\mathbf {z}},{\mathbf {s}})+{{\,\mathrm{excess}\,}}_{\sigma (i)}&= \max \{|s_i-z_i|,|z_i-z_{\sigma (i)}|\}+s_j-z_{\sigma (i)}\\&\ge \frac{1}{2} (z_{\sigma (i)}-s_i)+\frac{1}{2}(s_j-z_{\sigma (i)}) = \frac{1}{2}(s_j-s_i)\\&\ge \frac{1}{3}|s_i-s_{\eta (i)}| \end{aligned}$$

as desired. $$\square $$

### Claim 13.3

Let $i\in S$ such that $\sigma (i)\in L$ or $\sigma (i)\in {{{\mathcal {C}}}}\setminus {{{\mathcal {K}}}}$. Then, ${{\,\mathrm{cost}\,}}_i({\mathbf {z}},{\mathbf {s}})+{{\,\mathrm{cost}\,}}_{\sigma (i)}({\mathbf {z}},{\mathbf {s}}) \ge \frac{1}{3}(|s_i-s_{\eta (i)}|+|s_{\sigma (i)}-s_{\eta (\sigma (i))}|)$.

### Proof

We assume that $\sigma (i)>i$ (the other case is symmetric). If $z_{\sigma (i)}>s_{\sigma (i)}$, then

$$\begin{aligned} {{\,\mathrm{cost}\,}}_i({\mathbf {z}},{\mathbf {s}})&= \max \{|s_i-z_i|,|z_i-z_{\sigma (i)}|\}\\&\ge \frac{1}{2}(z_{\sigma (i)}-s_i) > \frac{1}{2}(s_{\sigma (i)}-s_i)\\&\ge \frac{1}{3}|s_i-s_{\eta (i)}|,\end{aligned}$$

which contradicts the fact that *i* is a small player. Hence, $z_{\sigma (i)}\in [s_i,s_{\sigma (i)}]$, otherwise player *i* would be covered. Then,

$$\begin{aligned} {{\,\mathrm{cost}\,}}_i({\mathbf {z}},{\mathbf {s}})+{{\,\mathrm{cost}\,}}_{\sigma (i)}({\mathbf {z}},{\mathbf {s}})&=\max \{|s_i-z_i|,|z_i-z_{\sigma (i)}|\}\\&\quad +\max \{|s_{\sigma (i)}-z_{\sigma (i)}|,|z_{\sigma (i)}-z_{\sigma (\sigma (i))}|\}\\&\ge z_{\sigma (i)}-z_i+s_{\sigma (i)}-z_{\sigma (i)}=s_{\sigma (i)}-z_i.\end{aligned}$$

Since *i* is small, we have $z_i<s_i+\frac{1}{3}(s_{\sigma (i)}-s_i)$ and we get

$$\begin{aligned} {{\,\mathrm{cost}\,}}_i({\mathbf {z}},{\mathbf {s}})+{{\,\mathrm{cost}\,}}_{\sigma (i)}({\mathbf {z}},{\mathbf {s}}) \ge \frac{2}{3}(s_{\sigma (i)}-s_i) \ge \frac{1}{3}|s_i-s_{\eta (i)}|+\frac{1}{3}|s_{\sigma (i)}-s_{\eta (\sigma (i))}| \end{aligned}$$

as desired. $$\square $$

Let *N*(*S*) denote the set of players *j* that are neighbors of players in *S* (i.e., $j\in N(S)$ when $\sigma (i)=j$ for some player $i\in S$).

### Claim 13.4

*N*(*S*) does not contain small players.

### Proof

Assume otherwise that for some player $i\in S$, $\sigma (i)$ also belongs to *S*. Without loss of generality $\sigma (i)>i$. If $z_{\sigma (i)}\ge s_{\sigma (i)}$, then

$$\begin{aligned} {{\,\mathrm{cost}\,}}_i({\mathbf {z}},{\mathbf {s}}) \ge \frac{1}{2}|z_{\sigma (i)}-s_i|\ge \frac{1}{2}{|s_{\sigma (i)}-s_i|} \ge \frac{1}{3}|s_i-s_{\eta (i)}| \end{aligned}$$

contradicting the fact that $i\in S$. So, $z_{\sigma (i)}<s_{\sigma (i)}$. Also, $z_{\sigma (i)}\ge s_i$ (since neither *i* is covered nor $\sigma (i)$ is kangaroo). Since $\sigma (i)$ is small, $s_{\sigma (i)}-z_{\sigma (i)}<\frac{1}{3}|s_{\sigma (i)}-s_{\eta (\sigma (i))}|\le \frac{1}{3}(s_{\sigma (i)}-s_i)$, i.e., $z_{\sigma (i)}>\frac{2}{3}s_{\sigma (i)}+\frac{1}{3}s_i$. Hence,

$$\begin{aligned} {{\,\mathrm{cost}\,}}_i({\mathbf {z}},{\mathbf {s}}) \ge \frac{1}{2}(z_{\sigma (i)}-s_i) > \frac{1}{3}(s_{\sigma (i)}-s_i), \end{aligned}$$

which contradicts $$i\in S$$. $$\square $$

### Claim 13.5

For every two players $i,i'\in S$, $\sigma (i)\not =\sigma (i')$.

### Proof

Assume otherwise and let $\sigma (i)=\sigma (i')=j$ with $i<i'$. If $z_j\not \in [s_i,s_{i'}]$, then the cost of either *i* or $i'$ is at least $\frac{1}{2}(s_{i'}-s_i)$, contradicting the fact that both players are small. Hence, $z_j\in [s_i,s_{i'}]$. Notice that $s_j\in [s_i,s_{i'}]$ as well, otherwise either *i* or $i'$ would be covered by *j*. Now the fact that *i* and $i'$ are small implies that

$$\begin{aligned}&{{\,\mathrm{cost}\,}}_i({\mathbf {z}},{\mathbf {s}})+{{\,\mathrm{cost}\,}}_{i'}({\mathbf {z}},{\mathbf {s}})<\frac{1}{3}|s_i-s_{\eta (i)}|\\&\qquad +\frac{1}{3}|s_{i'}-s_{\eta (i')}|\\&\quad \le \frac{1}{3}(s_j-s_i)+\frac{1}{3}(s_{i'}-s_j)=\frac{1}{3}(s_{i'}-s_i). \end{aligned}$$

On the other hand,

$$\begin{aligned} {{\,\mathrm{cost}\,}}_i({\mathbf {z}},{\mathbf {s}})+{{\,\mathrm{cost}\,}}_{i'}({\mathbf {z}},{\mathbf {s}})\ge \frac{1}{2}(z_j-s_i)+\frac{1}{2}(s_{i'}-z_j)=\frac{1}{2}(s_{i'}-s_i), \end{aligned}$$

a contradiction. $$\square $$

We now consider the social cost of ${\mathbf {z}}$ due to players of different groups and exploit the claims above so that we obtain the lemma. In particular, we have

$$\begin{aligned} {{\,\mathrm{\text {SC}}\,}}({\mathbf {z}},{\mathbf {s}})&= \sum _{i=1}^n{{{\,\mathrm{cost}\,}}_i({\mathbf {z}},{\mathbf {s}})}\\&\ge \sum _{i\in S:\sigma (i)\in {{{\mathcal {K}}}}}{\left( {{\,\mathrm{cost}\,}}_i({\mathbf {z}},{\mathbf {s}})+{{\,\mathrm{excess}\,}}_{\sigma (i)}\right) } \\&\quad + \sum _{i\in S:\sigma (i)\in L\cup ({{{\mathcal {C}}}}\setminus {{{\mathcal {K}}}})}{\left( {{\,\mathrm{cost}\,}}_i({\mathbf {z}},{\mathbf {s}})+{{\,\mathrm{cost}\,}}_{\sigma (i)}({\mathbf {z}},{\mathbf {s}})\right) }\\&\quad + \sum _{i\in {{{\mathcal {K}}}}}{\left( {{\,\mathrm{cost}\,}}_i({\mathbf {z}},{\mathbf {s}})-{{\,\mathrm{excess}\,}}_i\right) } + \sum _{i\in L\setminus N(S)}{{{\,\mathrm{cost}\,}}_i({\mathbf {z}},{\mathbf {s}})}\\&\ge \frac{1}{3}\sum _{i\in S:\sigma (i)\in {{{\mathcal {K}}}}}{|s_i-s_{\eta (i)}|} \\&\quad + \frac{1}{3}\sum _{i\in S:\sigma (i)\in L\cup ({{{\mathcal {C}}}}\setminus {{{\mathcal {K}}}})}{\left( |s_i-s_{\eta (i)}|+|s_{\sigma (i)}-s_{\eta (\sigma (i))}|\right) }\\&\quad + \frac{1}{3}\sum _{i\in {{{\mathcal {K}}}}}{\left( |s_i-s_{\eta (i)}|+\sum _{j\in C_i}{|s_j-s_{\eta (j)}|}\right) } + \frac{1}{3}\sum _{i\in L\setminus N(S)}{|s_i-s_{\eta (i)}|}\\&\ge \frac{1}{3}\sum _{i=1}^n{|s_i-s_{\eta (i)}|}, \end{aligned}$$

as desired. The first inequality follows by the classification of the players and due to Claims 13.4 and 13.5. The second one follows by Claims 13.2, 13.3, and 13.1, and by the definition of large players. The last one follows since the players enumerated in the first two sums at its left cover the whole set *S* (by Claim 13.4). $\square$

We are ready to present our upper bound on the price of anarchy for 1-COF games.

### Theorem 14

The price of anarchy of 1-COF games over pure Nash equilibria is at most 3.

### Proof

Let us consider a 1-COF game with *n* players and belief vector ${\mathbf {s}}$. Let ${\mathbf {z}}^*$ be an optimal opinion vector and recall that $\eta (i)$ is the player that minimizes the distance $|s_i-s_{\eta (i)}|$. By Lemma 13, we have

20 $$\begin{aligned} {{\,\mathrm{\text {SC}}\,}}({\mathbf {z}}^*,{\mathbf {s}})&\ge \frac{1}{3}\sum _{i=1}^n{|s_i-s_{\eta (i)}|}. \end{aligned}$$

Now, consider any pure Nash equilibrium ${\mathbf {z}}$ of the game. We will show that

21 $$\begin{aligned} {{\,\mathrm{\text {SC}}\,}}({\mathbf {z}},{\mathbf {s}})&\le \sum _{i=1}^n{|s_i-s_{\eta (i)}|}. \end{aligned}$$

The theorem then follows by (20) and (21).

In particular, we will show that ${{\,\mathrm{cost}\,}}_i({\mathbf {z}},{\mathbf {s}})\le |s_i-s_{\eta (i)}|$ for each player *i*. Let us assume that $\eta (i)=i-1$; the case $\eta (i)=i+1$ is symmetric. Recall that $\sigma (i)$ is the neighbor of player *i* in the pure Nash equilibrium ${\mathbf {z}}$. We distinguish between four cases.

- **Case I:** $\sigma (i)=i-1$. By Lemma 4, we have $s_{i-1}\le z_i\le s_i$. Then, clearly, ${{\,\mathrm{cost}\,}}_i({\mathbf {z}},{\mathbf {s}}) = |s_i-z_i| \le |s_i-s_{i-1}|$ as desired.
- **Case II:** $\sigma (i)=i+1$ and $\sigma (i-1)=i$. By Lemmas 2 and 4, we have $s_{i-1}\le z_{i-1}\le s_i \le z_i$. Since player *i* has player $i+1$ as neighbor, we have $|z_{i+1}-s_i|\le |s_i-z_{i-1}|$. Hence, ${{\,\mathrm{cost}\,}}_i({\mathbf {z}},{\mathbf {s}}) =|z_i-s_i|\le |z_{i+1}-s_i|\le |s_i-z_{i-1}|\le |s_i-s_{i-1}|$.
- **Case III:** $\sigma (i)=i+1$, $\sigma (i-1)=i-2$, and ${{\,\mathrm{cost}\,}}_i({\mathbf {z}},{\mathbf {s}})\le {{\,\mathrm{cost}\,}}_{i-1}({\mathbf {z}},{\mathbf {s}})$. By the definition of $\sigma (\cdot )$ and Lemma 2, we have $z_{i-2}\le z_{i-1}\le s_{i-1}\le s_i\le z_i\le z_{i+1}$. We have
  $$\begin{aligned} {{\,\mathrm{cost}\,}}_i({\mathbf {z}},{\mathbf {s}})&\le 2{{\,\mathrm{cost}\,}}_{i-1}({\mathbf {z}},{\mathbf {s}})-{{\,\mathrm{cost}\,}}_i({\mathbf {z}},{\mathbf {s}})\\&= |s_{i-1}-z_{i-2}|-|z_{i}-s_i|\\&\le |z_i-s_{i-1}|-|z_{i}-s_{i}|\\&= |s_i-s_{i-1}|. \end{aligned}$$
  The second inequality follows since player $i-2$ (instead of *i*) is the neighbor of player $i-1$.
- **Case IV:** $\sigma (i)=i+1$, $\sigma (i-1)=i-2$, and ${{\,\mathrm{cost}\,}}_i({\mathbf {z}},{\mathbf {s}})>{{\,\mathrm{cost}\,}}_{i-1}({\mathbf {z}},{\mathbf {s}})$.
  $$\begin{aligned} {{\,\mathrm{cost}\,}}_i({\mathbf {z}},{\mathbf {s}})&< 2{{\,\mathrm{cost}\,}}_i({\mathbf {z}},{\mathbf {s}})-{{\,\mathrm{cost}\,}}_{i-1}({\mathbf {z}},{\mathbf {s}})\\&= |z_{i+1}-s_i|-|s_{i-1}-z_{i-1}|\\&\le |s_i-z_{i-1}|-|s_{i-1}-z_{i-1}|\\&= |s_i-s_{i-1}|. \end{aligned}$$
  The second inequality follows since player $i+1$ (instead of $i-1$) is the neighbor of player *i*.

This completes the proof. $\square$

## Lower Bounds on the Price of Anarchy

This section contains our lower bounds on the price of anarchy.^3^ We begin by considering the simpler case of 1-COF games, for which we present a tight lower bound of 3 for pure Nash equilibria (Theorem 15) and a lower bound of 6 for mixed Nash equilibria (Theorem 16). We remark that, for 1-COF games, this implies that mixed Nash equilibria are strictly worse than pure ones. Then, we study the general case of *k*-COF games and we show lower bounds for pure and mixed Nash equilibria (Theorems 17 and 18, respectively) that grow linearly with *k*.

### The Case of 1-COF Games

We now present our lower bounds for the case of 1-COF games; both results rely on the same, and rather simple, instance.

**Fig. 9:**
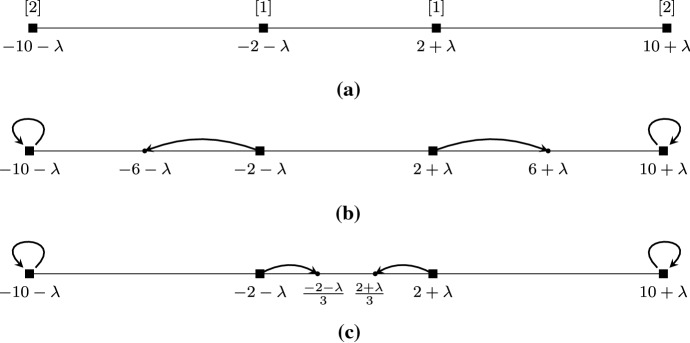
**a** The 1-COF game considered in the proofs of Theorems 15 and 16. **b** The pure Nash equilibrium vector ${\mathbf {z}}$ (see the proof of Theorem 15) with social cost 8. **c** The opinion vector ${\tilde{{\mathbf {z}}}}$ with social cost $\frac{8+4\lambda }{3}$

#### Theorem 15

The price of anarchy of 1-COF games over pure Nash equilibria is at least 3.

#### Proof

Let $\lambda \in (0,1)$ and consider a 1-COF game with six players and belief vector ${\mathbf {s}}=(-10-\lambda ,-10-\lambda ,-2-\lambda ,2+\lambda ,10+\lambda ,10+\lambda ).$ This game is depicted in Fig. 9a. We can show that the opinion vector (see Fig. 9b)

$$\begin{aligned} {\mathbf {z}}= (-10-\lambda ,-10-\lambda ,-6-\lambda ,6+\lambda ,10+\lambda ,10+\lambda ) \end{aligned}$$

is a pure Nash equilibrium with social cost ${{\,\mathrm{\text {SC}}\,}}({\mathbf {z}}, {\mathbf {s}}) = 8$. The first two players suffer zero cost as they follow each other and their opinions coincide with their beliefs; the same holds also for the last two players. For the third player, it is $\sigma (3) \in \{1,2\}$ since $|z_1-s_3| = |z_2-s_3| = 8 < |z_4-s_3| = 8+2\lambda$ and $z_3$ is in the midpoint of the interval $[-10-\lambda ,-2-\lambda ]$; hence, ${{\,\mathrm{cost}\,}}_3({\mathbf {z}},{\mathbf {s}}) = 4$. Similarly, we have $\sigma (4) \in \{5,6\}$, $z_4$ lies in the midpoint of the interval $[2+\lambda , 10+\lambda ]$ and ${{\,\mathrm{cost}\,}}_4({\mathbf {z}},{\mathbf {s}}) = 4$. Hence, ${\mathbf {z}}$ is indeed a pure Nash equilibrium.

Now, consider the opinion vector (see Fig. 9c)

$$\begin{aligned} {\tilde{{\mathbf {z}}}} = \left( -10-\lambda ,-10-\lambda ,\frac{-2-\lambda }{3},\frac{2+\lambda }{3},10+\lambda ,10+\lambda \right) \end{aligned}$$

which yields a social cost of ${{\,\mathrm{\text {SC}}\,}}({\tilde{{\mathbf {z}}}}, {\mathbf {s}}) = \frac{8+4\lambda }{3}$; here, again, the first and last two players have zero cost, but players 3 and 4 now each have cost $\frac{4+2\lambda }{3}$ since they follow each other. The optimal social cost is upper bounded by ${{\,\mathrm{\text {SC}}\,}}({\tilde{{\mathbf {z}}}})$ and, hence, the price of anarchy is at least

$$\begin{aligned} \frac{{{\,\mathrm{\text {SC}}\,}}({\mathbf {z}}, {\mathbf {s}})}{{{\,\mathrm{\text {SC}}\,}}({\tilde{{\mathbf {z}}}}, {\mathbf {s}})} = \frac{3}{1+\lambda /2}, \end{aligned}$$

and the theorem follows by setting $\lambda$ arbitrarily close to 0. $\square$

Our next theorem gives a lower bound on the price of anarchy over mixed Nash equilibria for 1-COF games; we remark that this lower bound is greater than the upper bound of Theorem 14 for the price of anarchy over pure Nash equilibria.

#### Theorem 16

The price of anarchy of 1-COF games over mixed Nash equilibria is at least 6.

#### Proof

Consider again the 1-COF game depicted in Fig. 9a with six players and belief vector ${\mathbf {s}}=(-10-\lambda ,-10-\lambda ,-2-\lambda ,2+\lambda ,10+\lambda ,10+\lambda )$, where $\lambda \in (0,1)$. To simplify the following discussion, we will refer to the first two players as the *L* players, the third player as player $\ell$, the fourth player as player *r*, and the last two players as the *R* players.

Let ${\mathbf {z}}$ be a randomized opinion vector according to which $z_i=s_i$ for every $i \in L \cup R$, $z_\ell$ is chosen equiprobably from $\{-6-\lambda , -6+3\lambda \}$, and $z_r$ is chosen equiprobably from $\{6+\lambda , 6-3\lambda \}$. Observe that $\sigma (\ell ) \in L$ whenever $z_r = 6+\lambda$, and $\sigma (\ell ) = r$ whenever $z_r = 6-3\lambda$; each of these events occurs with probability 1/2. Hence, we obtain

$$\begin{aligned}&{\mathbb {E}}[{{\,\mathrm{cost}\,}}_{\ell }({\mathbf {z}},{\mathbf {s}})] = {\mathbb {E}}[{{\,\mathrm{cost}\,}}_r({\mathbf {z}},{\mathbf {s}})] = \frac{1}{2}\left( \frac{4}{2} +\frac{4+4\lambda }{2}\right) \\&\quad +\frac{1}{2}\left( \frac{12-2\lambda }{2}+\frac{12-6\lambda }{2}\right) = 8-\lambda , \end{aligned}$$

and, thus, ${\mathbb {E}}[{{\,\mathrm{\text {SC}}\,}}({\mathbf {z}},{\mathbf {s}})] = 16-2\lambda$. In the following, we will prove that ${\mathbf {z}}$ is a mixed Nash equilibrium. First, observe that all players in sets *L* and *R* have no incentive to deviate since they follow each other and have zero cost. We will now argue about player $\ell$; due to symmetry, our findings will apply to player *r* as well.

Consider a deterministic deviating opinion *y* for player $\ell$. We will show that ${\mathbb {E}}[{{\,\mathrm{cost}\,}}_{\ell }({\mathbf {z}},{\mathbf {s}})]\le {\mathbb {E}}_{{\mathbf {z}}_{-\ell }}[{{\,\mathrm{cost}\,}}_{\ell }(y,{\mathbf {z}}_{-\ell }),{\mathbf {s}}]$ for any *y*, which implies that player $\ell$ has no incentive to deviate from the randomized opinion $z_\ell$. Indeed, we have that

$$\begin{aligned}&{\mathbb {E}}_{{\mathbf {z}}_{-\ell }}[{{\,\mathrm{cost}\,}}_\ell ((y,{\mathbf {z}}_{-\ell }),{\mathbf {s}})] \\&= \frac{1}{2}\max \{|-2-\lambda -y|,|y+10+\lambda |\}+\frac{1}{2}\max \{|-2-\lambda -y|,|6-3\lambda -y|\}\\&\ge \frac{1}{2}(y+10+\lambda )+\frac{1}{2}(6-3\lambda -y)\\&= 8-\lambda , \end{aligned}$$

where the inequality holds since $\max \{|a|,|b|\}\ge a$ for any *a* and *b*. Hence, player $\ell$ has no incentive to deviate from her strategy in ${\mathbf {z}}$, and neither has player *r* due to symmetry. Therefore, ${\mathbf {z}}$ is a mixed Nash equilibrium.

Now, consider the opinion vector

$$\begin{aligned} {\tilde{{\mathbf {z}}}} = \left( -10-\lambda ,-10-\lambda ,\frac{-2-\lambda }{3},\frac{2+\lambda }{3},10+\lambda ,10+\lambda \right) \end{aligned}$$

which, as in Theorem 15, yields a social cost of ${{\,\mathrm{\text {SC}}\,}}({\tilde{{\mathbf {z}}}}, {\mathbf {s}}) = \frac{8+4\lambda }{3}$. Hence, the optimal social cost is upper bounded by ${{\,\mathrm{\text {SC}}\,}}({\tilde{{\mathbf {z}}}}, {\mathbf {s}})$, and the price of anarchy over mixed equilibria is at least

$$\begin{aligned} \frac{{\mathbb {E}}[{{\,\mathrm{\text {SC}}\,}}({\mathbf {z}}, {\mathbf {s}})]}{{{\,\mathrm{\text {SC}}\,}}({\tilde{{\mathbf {z}}}}, {\mathbf {s}})} = 3\frac{16-2\lambda }{8+4\lambda }, \end{aligned}$$

and the theorem follows by setting $\lambda$ arbitrarily close to 0. $\square$

### The General Case of *k*-COF Games with $k\ge 2$

We will now present lower bounds on the price of anarchy for *k*-COF games, with $k\ge 2$. We start with the case of pure Nash equilibria and continue with the more general case of mixed equilibria. As in the case of 1-COF games, a particular game will be used in order to derive the lower bounds both for pure and mixed Nash equilibria.

**Fig. 10:**
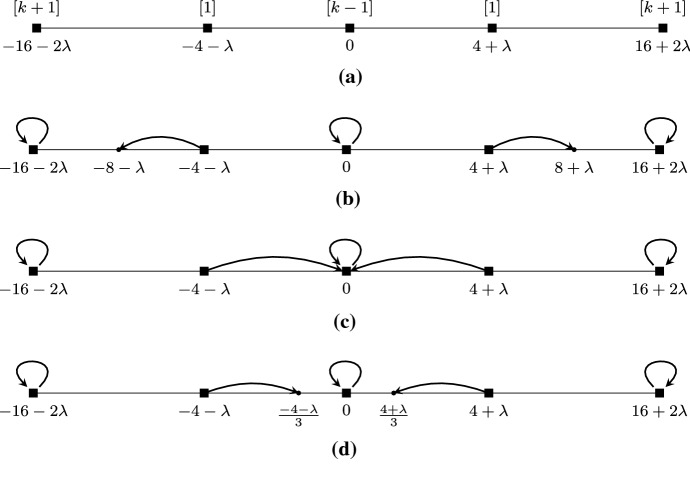
**a** The *k*-COF game considered in the proofs of Theorems 17 and 18, for $k\ge 2$. **b** The pure Nash equilibrium opinion vector ${\mathbf {z}}$ (see the proof of Theorem 17). **c** The optimal opinion vector ${\tilde{{\mathbf {z}}}}$ for $k\ge 3$. **d** The optimal opinion vector ${\tilde{{\mathbf {z}}}}$ for $k=2$. Observe that the optimal opinion vector changes at $k=2$ due to the neighborhood size

#### Theorem 17

The price of anarchy of *k*-COF games over pure Nash equilibria is at least $k+1$ for $k \ge 3$, and at least 18/5 for $k=2$.

#### Proof

Let $\lambda \in (0,1)$ and consider a *k*-COF game with $3k+3$ players, for $k\ge 2$, that are partitioned into the following five sets. The first set *L* consists of $k+1$ players with $s_i = -16-2\lambda$ for any $i\in L$, the second set consists of a single player $\ell$ with $s_{\ell } = -4-\lambda$, the third set *M* has $k-1$ players with $s_i = 0$ for any $i\in M$, the fourth set is a single player *r* with $s_r = 4+\lambda$, and the last set *R* consists of $k+1$ players with $s_i= 16+2\lambda$ for any $i\in R$. This instance is depicted in Fig. 10a.

Let ${\mathbf {z}}$ be the following opinion vector: $z_i=-16-2\lambda$ for any $i \in R$, $z_\ell = -8-\lambda$, $z_i = 0$ for any $i \in M$, $z_r = 8+\lambda$, and $z_i = 16+2\lambda$ for any $i \in R$; see Fig. 10b. It is not hard to verify that this opinion vector is a pure Nash equilibrium with social cost ${{\,\mathrm{\text {SC}}\,}}({\mathbf {z}},{\mathbf {s}}) = (8+\lambda )(k+1)$. First, observe that all players in sets *L* and *R* have zero cost, and, hence, have no incentive to deviate to another opinion. Furthermore, no player $i \in M$ has an incentive to deviate either since $z_i$ lies in the midpoint of the interval $[-8-\lambda ,8+\lambda ]$ which is defined by the opinions of players $\ell$ and *r* who, together with the remaining players of *M*, constitute the neighborhood $N_i({\mathbf {z}},{\mathbf {s}})$ of player *i*. The cost experienced by such a player *i* is $8+\lambda$. Finally, the neighborhood $N_\ell ({\mathbf {z}},{\mathbf {s}})$ of player $\ell$ consists of all players in *M* (who have opinions that are closest to $s_\ell$) and some player $i \in L$; note that player *r* does not belong to $N_\ell ({\mathbf {z}},{\mathbf {s}})$ since $z_r - s_\ell = 12+2\lambda > 12-\lambda = s_\ell -z_i$ for all $i \in L$. Hence, player $\ell$ has no incentive to deviate to another opinion since $z_\ell$ lies in the midpoint of the interval $[-16-2\lambda ,0]$ and she experiences cost equal to $8+\lambda$. Due to symmetry, player *r* does not have incentive to deviate as well. Hence, ${\mathbf {z}}$ is indeed a pure Nash equilibrium with ${{\,\mathrm{\text {SC}}\,}}({\mathbf {z}},{\mathbf {s}}) = (8+\lambda )(k+1)$.

We now present an opinion vector ${\tilde{{\mathbf {z}}}}$ with social cost ${{\,\mathrm{\text {SC}}\,}}({\tilde{{\mathbf {z}}}},{\mathbf {s}}) = 8+2\lambda$ for $k\ge 3$ and ${{\,\mathrm{cost}\,}}({\tilde{{\mathbf {z}}}},{\mathbf {s}}) = \frac{5}{3}(4+\lambda )$ for $k=2$. In particular, for $k\ge 3$, ${\tilde{{\mathbf {z}}}}$ is defined as follows: ${\tilde{z}}_i = -16-2\lambda$ for any $i \in L$, ${\tilde{z}}_\ell = {\tilde{z}}_i = {\tilde{z}}_r = 0$ for any $i \in M$, and ${\tilde{z}}_i = 16+2\lambda$ for any $i \in R$; see Fig. 10c. Observe that all players in *L*, *M*, and *R* have zero cost, while players $\ell$ and *r* have cost equal to $4+\lambda$ each. For $k=2$, ${\tilde{{\mathbf {z}}}}$ is defined as follows: ${\tilde{z}}_i = -16-2\lambda$ for any $i \in L$, ${\tilde{z}}_\ell = -\frac{1}{3}(4+\lambda )$, ${\tilde{z}}_i = 0$ for any $i \in M$, ${\tilde{z}}_r = \frac{1}{3}(4+\lambda )$, and ${\tilde{z}}_i = 16+2\lambda$ for any $i \in R$; see Fig. 10d. Again, all players in *L* and *R* have zero cost. However, players $\ell$ and *r* now each have cost $\frac{2}{3}(4+\lambda )$ and the unique player in *M* has cost $\frac{1}{3}(4+\lambda )$.

Clearly, since ${{\,\mathrm{\text {SC}}\,}}({\tilde{{\mathbf {z}}}},{\mathbf {s}})$ is an upper bound on the optimal social cost, we conclude that the price of anarchy over pure Nash equilibria is at least $\frac{(8+\lambda )(k+1)}{8+2\lambda }$ for $k\ge 3$ and $\frac{9(8+\lambda )}{5(4+\lambda )}$ for $k=2$, and the theorem follows by setting $\lambda$ arbitrarily close to 0. $\square$

We now consider the case of mixed Nash equilibria; we remark that, in this case, our lower bounds for $k\ge 2$ are smaller than the corresponding upper bounds for pure Nash equilibria.

#### Theorem 18

The price of anarchy of *k*-COF games over mixed Nash equilibria is at least $k+2$ for $k \ge 3$, and at least 24/5 for $k=2$.

#### Proof

As in the proof of Theorem 17, let $\lambda \in (0,1)$ and consider the *k*-COF game depicted in Fig. 10a with $3k+3$ players that form 5 sets. Again, the first set *L* consists of $k+1$ players where $s_i = -16-2\lambda$ for all $i\in L$, the second set consists of a single player $\ell$ with $s_{\ell } = -4-\lambda$, the third set *M* has $k-1$ players with $s_i = 0$ for all $i\in M$, the fourth set is a single player *r* with $s_r = 4+\lambda$, and the last set *R* consists of $k+1$ players with $s_i = 16+2\lambda$ for all $i\in R$.

Consider the following (randomized) opinion vector ${\mathbf {z}}$: $z_i = s_i$ for every $i \in L\cup M\cup R$, while $z_\ell$ is chosen uniformly at random among $\{-8-\lambda , -8+3\lambda \}$ and $z_r$ is chosen uniformly at random among $\{8-3\lambda ,8+\lambda \}$. We will show that the opinion vector ${\mathbf {z}}$ is a mixed Nash equilibrium with ${\mathbb {E}}[{{\,\mathrm{\text {SC}}\,}}({\mathbf {z}}, {\mathbf {s}})] = 8k+16-\lambda$.

First, observe that the players in sets *L* and the *R* constitute local neighborhoods, that is, $N_i({\mathbf {z}},{\mathbf {s}}) = L\setminus \{i\}$ for any player $i \in L$, and $N_i({\mathbf {z}},{\mathbf {s}})=R\setminus \{i\}$ for any player $i \in R$. Hence, all these players have zero cost and no incentive to deviate.

Next, let us focus on a player $i \in M$. Clearly, the neighborhood of player *i* consists of the remaining $k-2$ players in *M* as well as players $\ell$ and *r*. The expected cost of player *i* in ${\mathbf {z}}$ is ${\mathbb {E}}[{{\,\mathrm{cost}\,}}_i({\mathbf {z}},{\mathbf {s}})] = \frac{3}{4}(8+\lambda )+\frac{1}{4}(8-3\lambda ) = 8$ since at least one of players $\ell$ and *r* is at distance $8+\lambda$ with probability 3/4 and both of them are at distance $8-3\lambda$ with probability 1/4. Hence, these $k-1$ players contribute $8(k-1)$ to the expected social cost of ${\mathbf {z}}$. We now argue that if player $i \in M$ deviates to a deterministic opinion *y*, her expected cost does not decrease. Clearly, if $y\ge 3\lambda$, then this trivially holds as the expected cost of *i* is at least $y-z_\ell$ which is at least $y+8-3\lambda$; the case where $y\le -3\lambda$ is symmetric. Hence, it suffices to consider the case where $|y|< 3\lambda$. The expected cost of *i* when deviating to *y* is

$$\begin{aligned}&{\mathbb {E}}_{{\mathbf {z}}_{-i}}[{{\,\mathrm{cost}\,}}_i((y,{\mathbf {z}}_{-i}),{\mathbf {s}})] \\&= \frac{1}{4}\max \{8+\lambda -y,y+8+\lambda \}+\frac{1}{4}\max \{8+\lambda -y,y+8-3\lambda \}\\&\quad + \frac{1}{4}\max \{8-3\lambda -y,y+8+\lambda \} +\frac{1}{4}\max \{8-3\lambda -y,y+8-3\lambda \}\\&\ge \frac{1}{4}(8+\lambda -y)+\frac{1}{4}(8+\lambda -y)+\frac{1}{4}(y+8+\lambda )+\frac{1}{4}(y+8-3\lambda )\\&= 8, \end{aligned}$$

where the inequality holds since $\max \{a,b\}\ge a$ for any *a* and *b*.

Now, let us examine player *r*; the case of player $\ell$ is symmetric. Observe that the $k-1$ players in *M* always belong to the neighborhood $N_r({\mathbf {z}},{\mathbf {s}})$ of player *r* and it remains to argue about the identity of the last player in $N_r({\mathbf {z}},{\mathbf {s}})$. Whenever $z_\ell = -8+3\lambda$, then $\ell \in N_r({\mathbf {z}},{\mathbf {s}})$, otherwise, if $z_\ell = -8-\lambda$, one of the players in set *R* belongs to $N_r({\mathbf {z}},{\mathbf {s}})$. The expected cost of player *r* is ${\mathbb {E}}[{{\,\mathrm{cost}\,}}_r({\mathbf {z}},{\mathbf {s}})] = \frac{1}{4}(8+\lambda ) + \frac{1}{4}(8+5\lambda )+\frac{1}{4}(16-2\lambda )+\frac{1}{4}(16 - 6\lambda )= 12-\lambda /2$, and, hence, players $\ell$ and *r* contribute $24-\lambda$ to the expected social cost of ${\mathbf {z}}$. It remains to show that player *r* cannot decrease her expected cost by deviating to another opinion *y*. The expected cost of player *r* when deviating to *y* is

$$\begin{aligned} {\mathbb {E}}_{{\mathbf {z}}_{-r}}[{{\,\mathrm{cost}\,}}_r((y,{\mathbf {z}}_{-r}),{\mathbf {s}})]&= \frac{1}{2}\max \{|16+2\lambda -y|,|y|\}\\&+\frac{1}{2}\max \{|y+8-3\lambda |,|4+\lambda -y|\}\\&\ge \frac{1}{2}(16+2\lambda -y)+\frac{1}{2}(y+8-3\lambda )\\&= 12-\lambda /2, \end{aligned}$$

where the inequality holds since $\max \{|a|,|b|\}\ge a$ for any *a* and *b*. Hence, we conclude that ${\mathbf {z}}$ is a mixed Nash equilibrium with expected social cost ${\mathbb {E}}[{{\,\mathrm{\text {SC}}\,}}({\mathbf {z}},{\mathbf {s}})] = 8k+16-\lambda$.

As in the proof of Theorem 17, there exists an opinion vector ${\tilde{{\mathbf {z}}}}$ with social cost ${{\,\mathrm{\text {SC}}\,}}({\tilde{{\mathbf {z}}}},{\mathbf {s}}) = 8+2\lambda$ for $k\ge 3$ and ${{\,\mathrm{\text {SC}}\,}}({\tilde{{\mathbf {z}}}},{\mathbf {s}}) = \frac{5}{3}(4+\lambda )$ for $k=2$. Since ${{\,\mathrm{\text {SC}}\,}}({\tilde{{\mathbf {z}}}},{\mathbf {s}})$ is an upper bound on the optimal social cost, we have that the price of anarchy over mixed equilibria is at least $\frac{8k+16-\lambda }{8+2\lambda }$ for $k\ge 3$ and $\frac{3(32-\lambda )}{5(4+\lambda )}$ for $k=2$, and the theorem follows, again by setting $\lambda$ arbitrarily close to 0. $\square$

## Open Problems and Extensions

We have introduced the class of compromising opinion formation games by enriching coevolutionary opinion games with a cost function that urges players to “meet halfway”. Our findings indicate that the quality of their equilibria deteriorates as the neighborhood size increases; in particular, the price of anarchy and the price of stability are $\varTheta (k)$. Still, there exists a gap between our lower and upper bounds for $k\ge 2$ and closing this gap is a challenging technical task. Furthermore, we know that mixed equilibria are strictly worse for 1-COF games but we have been unable to prove upper bounds on their price of anarchy. Is their price of anarchy still linear?

Another natural question is about the complexity of pure Nash equilibria in *k*-COF games for $k\ge 2$. We conjecture that there exists a polynomial time algorithm for computing them, but finding such an algorithm remains elusive. Similarly, what is the complexity of computing an optimal opinion vector (even for $k=1$)?

Finally, our modeling assumption that the number of neighbors is the same for all players is rather restrictive. Extending our results to the general case of different neighborhood size per player deserves investigation. One possible such generalization is to combine our approach to the Hegselmann-Krause model, i.e., each player’s neighborhood consists solely of those players with opinions sufficiently close to her belief.

## Footnotes

1 Note that we have proved monotonicity of opinions for pure Nash equilibria only (Lemma 2) and it is not clear whether such a monotonicity property holds for opinion vectors of minimum social cost. In addition, the statement of Lemma 11 refers to any opinion vector. This clearly includes non-monotonic ones, so we need to rank players in terms of opinions in the proof.

2 Case I cannot appear when $k=1$.

3 We remark that our lower bounds on the price of stability in Sect. 4 are also lower bounds on the price of anarchy. However, the lower bounds presented in this section are much stronger.
